# High-performance piezoelectric composites via β phase programming

**DOI:** 10.1038/s41467-022-32518-3

**Published:** 2022-08-18

**Authors:** Yuanjie Su, Weixiong Li, Xiaoxing Cheng, Yihao Zhou, Shuai Yang, Xu Zhang, Chunxu Chen, Tiannan Yang, Hong Pan, Guangzhong Xie, Guorui Chen, Xun Zhao, Xiao Xiao, Bei Li, Huiling Tai, Yadong Jiang, Long-Qing Chen, Fei Li, Jun Chen

**Affiliations:** 1 State Key Laboratory of Electronic Thin Films and Integrated Devices, School of Optoelectronic Science and Engineering, University of Electronic Science and Technology of China, 610054 Chengdu, China; 2 Department of Materials Science and Engineering, The Pennsylvania State University, State College, PA 16802 USA; 3 Department of Bioengineering, University of California, Los Angeles, Los Angeles, CA 90095 USA; 4 Electronic Materials Research Lab, Key Lab of Education Ministry/International Center for Dielectric Research, School of Electronic and Information Engineering, State Key Laboratory for Mechanical Behavior of Materials, Xi’an Jiaotong University, 710049 Xi’an, China; 5 School of Materials Science and Engineering, Research Center for Materials Genome Engineering, Wuhan University of Technology, 430070 Wuhan, China

**Keywords:** Two-dimensional materials, Electrical and electronic engineering

## Abstract

Polymer-ceramic piezoelectric composites, combining high piezoelectricity and mechanical flexibility, have attracted increasing interest in both academia and industry. However, their piezoelectric activity is largely limited by intrinsically low crystallinity and weak spontaneous polarization. Here, we propose a Ti_3_C_2_T_x_ MXene anchoring method to manipulate the intermolecular interactions within the all-*trans* conformation of a polymer matrix. Employing phase-field simulation and molecular dynamics calculations, we show that OH surface terminations on the Ti_3_C_2_T_x_ nanosheets offer hydrogen bonding with the fluoropolymer matrix, leading to dipole alignment and enhanced net spontaneous polarization of the polymer-ceramic composites. We then translated this interfacial bonding strategy into electrospinning to boost the piezoelectric response of samarium doped Pb (Mg_1/3_Nb_2/3_)O_3_-PbTiO_3_/polyvinylidene fluoride composite nanofibers by 160% via Ti_3_C_2_T_x_ nanosheets inclusion. With excellent piezoelectric and mechanical attributes, the as-electrospun piezoelectric nanofibers can be easily integrated into the conventional shoe insoles to form a foot sensor network for all-around gait patterns monitoring, walking habits identification and Metatarsalgi prognosis. This work utilizes the interfacial coupling mechanism of intermolecular anchoring as a strategy to develop high-performance piezoelectric composites for wearable electronics.

## Abstract (web summary)

The piezoelectricity of PVDF composites is mainly determined by the crystalline phases and spontaneous polarization. Here, the authors propose a Ti_3_C_2_T_x_ anchoring method to modulate the molecular interactions and conformation of polymer matrix.

## Introduction

Piezoelectric materials are critical to develop advanced sensors and actuators^1–7^. Due to the intriguing attributes of light weight, flexibility, biocompatibility and easy processibility, polyvinylidene fluoride (PVDF) and its copolymers have been widely employed in the fields of sensing, transducing and energy applications^8–13^. The piezoelectricity of PVDF composites is mainly determined by the crystalline phases and spontaneous polarization, where the α phase has TGTG′ (T-*trans*, G-*gauche*^+^, G’-*gauche*^*-*^) dihedral conformation with a dihedral angle of ±60°, while the β phase has all-*trans* (TTTT) conformation with a dihedral angle of 180° and the γ phase has kinked conformation of TTTGTTG′. Among these, the β phase is the most electroactive polar phase, exhibiting excellent piezoelectric, pyroelectric and ferroelectric properties^14^. Accordingly, obtaining the β phase is essential for improving the piezoelectricity of fluoropolymers. To this end, various processing methods were utilized to induce dipole alignment for increasing the electroactive β phase content including mechanical stretching^15^, electric poling^16^ and thermal annealing^17^. Meanwhile, the inclusion of TrFE monomers into PVDF chains can also modify the crystallization kinetics of PVDF films and lead to morphotropic phase boundary (MPB)-like behaviors (e.g., the high piezoelectric activity) in piezoelectric polymers^8,11,18–20^. By synergizing the merits of both high-performance piezoelectric ceramics and flexible fluoropolymers, the piezoelectric composites are the choice of materials for next-generation on-body bioelectronics toward widespread scenarios, such as soft robotics^21^, biomonitoring^22–26^, and human-machine interface^5,13,27^. Nevertheless, the random distribution and complex connectivity of ceramic fillers within the fluoropolymer matrix cause inhomogeneity and discontinuity of inorganic-organic interfaces, which hampers the long-range alignment of dipole moments in the polymeric chains and thus the all-*trans* conformation (i.e., polar β-phase)^28–30^. Meanwhile, the large discrepancy in dielectric permittivity between ceramic fillers and polymer matrix dramatically weakens the applied electric field in the ceramics during electric poling and thereby restricts the domain evolution and permanent polarization^31–33^. Consequently, it is highly desired to develop high-performance piezoelectric composites with long-range all-*trans* conformation and strong polarization.

In order to promote and stabilize the ratio of the polar β-phase, versatile fillers such as carbon nanotubes^34^, silver nanowires (AgNWs)^6,35^, ferrites^36–39^, graphene oxide (GO) and reduced graphene oxide (rGO) salts^40–42^ have been introduced as a nucleating agent to maximize and maintain the zigzag alignment of methylene (−CH_2_) and difluoromethylene (−CF_2_) groups within the polymer chains. Note that functional groups of the GOs and their derivates are conducive to forming dipole moment reinforcements to sustain the residual orientation of β-phase nanocrystals^40^. Meanwhile, the essential factor for the nucleation of the β-phase in the PVDF nanocomposites lies in the static electric interaction between the fillers with a negative zeta potential and the −CH_2_ groups having a positive charge density^35–38^. Despite the formation of β-phase could be facilitated partially by doping nanofillers, the sparse functional groups, discontinuous interface, low compatibility and inability to align domain on a large-scale of conductive fillers make it rather challenging to attain a homogeneous and long-range molecular interaction with PVDF molecular chains^43,44^, which significantly hinders the dipole polarization and thus the all-*trans* conformation of the as-synthesized polymer composites. Meanwhile, the mechanism of domain alignment enabled by intermolecular interaction at the inorganic-organic interfaces in piezoelectric composites is still unclear and underexplored.

Herein, we demonstrate a simple and efficient strategy for tailoring the local dipole moment and β phase content of piezoelectric polymer composites by introducing Ti_3_C_2_T_x_ MXene nanosheets. Employing phase-field simulation and molecular dynamics (MD) calculations, we show that OH surface terminations on the Ti_3_C_2_T_x_ nanosheets offer hydrogen bonding with the fluoropolymer matrix, leading to dipole alignment and enhanced net spontaneous polarization of the polymer-ceramic composites. We then translated this interfacial bonding method into electrospinning to rationalize the piezoelectric properties of samarium doped PMN-PT/PVDF (Sm-PMN-PT/PVDF) nanofibers via Ti_3_C_2_T_x_ nanosheets inclusion. It was found that an appropriate loading (2.5 wt%) of Ti_3_C_2_T_x_ flakes below the percolation threshold effectively strengthen the polarization efficiency and interfacial coupling between the inorganic nanofillers and the organic polymer matrix, promoting the piezoelectricity by 160 % in comparison with the undoped version. Toward practical application, a soft piezoelectric textile (PT) sensor was developed by electrospinning the MXene-enabled piezoelectric composite (MPC), which can be integrated into shoe insole for continuous gait patterns monitoring, walking habits identification, and Metatarsalgia prognosis.

## Results

### MD simulation of Ti_3_C_2_T_x_ enabled intermolecular anchoring effect

**Fig. 1:**
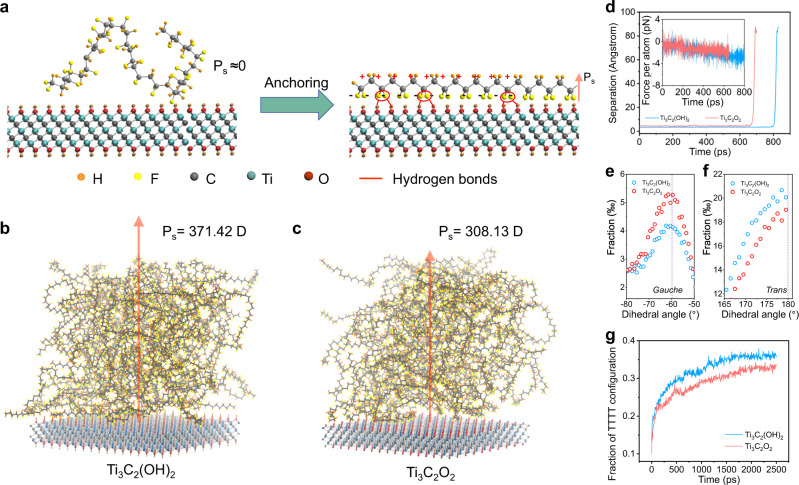
Hydrogen bonds induced anchoring effect enabled by MXene nanosheets. **a** Schematic of in-situ stretching and alignment of PVDF polymer chain via surface terminations on Ti_3_C_2_T_x_ nanosheets to upgrade the spontaneous polarization (P_s_). **b**, **c** Final snapshots for molecular dynamics (MD) simulations of the polarization of PVDF polymer film on the Ti_3_C_2_(OH)_2_ flakes (**b**) and Ti_3_C_2_O_2_ flakes (**c**). **d** Simulated separation of a single PVDF chain away from Ti_3_C_2_(OH)_2_ and Ti_3_C_2_O_2_ flakes upon pulling. Inset: The pulling force applied on each atom needed to separate the PVDF chain from MXene flakes. **e**, **f** Dihedral angles distribution of PVDF chains near the −60° (*Gauche*, **e**) and 180° (*Trans*, **f**) averaged over the last 500 ps. **g** Fraction of TTTT configuration as a function of time.

Ti_3_C_2_T_x_ nanosheets were exfoliated from the Ti_3_AlC_2_ MAX phase by selectively etching Al layers using a HCl/LiF solution, during which the surface transition metal spontaneously reacts with water or fluoride ions to produce hydroxyl (−OH), oxygen (−O), and fluoro (−F) surface terminations. These abundant functional groups terminated on the Ti_3_C_2_T_x_ lamellae arising from chemical etching are favorable for designing hydrogen, ionic and covalent linkage with surrounding dielectrics^45–47^. To this end, Ti_3_C_2_T_x_ nanosheets were employed to establish hydrogen bonds and electron-dipole interaction with C−H and C−F moieties of the PVDF molecules, enabling strong intermolecular bindings with PVDF polymer chains as shown in Fig. 1a. The local anchoring of PVDF chains on the two-dimensional MXene platelets directs in-situ alignment and orientation of CH_2_ and CF_2_ moieties and therefore transition from initial randomly coiled conformations (left part of Fig. 1a) to long-range all-*trans* conformation (right part of Fig. 1a), enlarging the macroscopic out-of-plane polarization and piezoelectricity.

To understand the interaction between the Ti_3_C_2_T_x_ nanosheets and fluoropolymer, MD calculations were implemented using the periodic lattice of Ti_3_C_2_T_x_ with −OH surface terminations and 60 “mer” chains of PVDF (Fig. 1b). To better illustrate the functionality and mechanism of hydrogen bonds between −OH group and −CF_2_ moieties of the PVDF, the corresponding MXene flakes functionalized with −O termination (Fig. 1c) were also constructed and calculated with the same number of “mer” chains. Apparently, after 2000 ps interaction, the PVDF chains anchored on the Ti_3_C_2_(OH)_2_ lamellae accomplish an out-of-plane polarization of 371.42 D under electrical poling while the counterpart versions on the Ti_3_C_2_O_2_ platelets trigger an out-of-plane polarization of 308.13 D (Supplementary Fig. 1), suggesting the enhancement of self-assembly of highly aligned PVDF chains on the Ti_3_C_2_(OH)_2_ flakes via the intermolecular interaction enabled by hydrogen bonding. Additionally, the pulling force needed to apply on each atom of the single PVDF chain is respectively 2.919 pN and 1.807 pN for Ti_3_C_2_(OH)_2_ and Ti_3_C_2_O_2_ to initialize the separation (Fig. 1d), implying a stronger interaction and tighter locking between Ti_3_C_2_(OH)_2_ flakes and PVDF chains. Without hydroxyl groups to guide the alignment of moieties of the PVDF molecule, the dipoles within the PVDF matrix would present irregular distribution to minimize the internal electrostatic energy (Supplementary Figs. 2, 3).

The phase transition could be clearly reflected from the variation of dihedral angles in bond conformation within the PVDF chains, where the most favorable torsional bond arrangement has substituents at 180° (*trans* or T) to each other instead of the ones at ±60° (*gauche* or G)^14^. Notably, the −OH functionalized Ti_3_C_2_T_x_ platelets possess a higher proportion of bond conformation near 180° (Fig. 1f) but a lower fraction of bond conformation near −60° (Fig. 1e and Supplementary Fig. 4) vice versa, indicating the transform from the *gauche* conformation to *trans* conformation as a consequence of hydrogen bonding induced anchoring effect. Interestingly, according to the MD calculation, Ti_3_C_2_(OH)_2_ platelets give rise to a higher fraction of T bond fraction (Supplementary Fig. 5) and TTTT (all-*trans*) conformation (Fig. 1g) in the PVDF chains in comparison with that of Ti_3_C_2_O_2_, which validates the role and functionality of hydrogen bonds in terms of arrangement and orientation of polymer chains toward all-*trans* conformation (i.e., polar β phase), promoting the net spontaneous polarization and the piezoelectricity of the fluoropolymer composites. Aside from the PVDF, the β-phase content and polarization of poly(vinylidene fluoride-co-trifluoroethylene) (P(VDF-TrFE)) copolymers can also be enhanced by this proposed MXene anchoring method according to the MD simulation (Supplementary Figs. 6, 7), validating the universality and feasibility.

### Design of MXene/Sm-PMN-PT/PVDF nonwoven piezoelectric composites

**Fig. 2:**
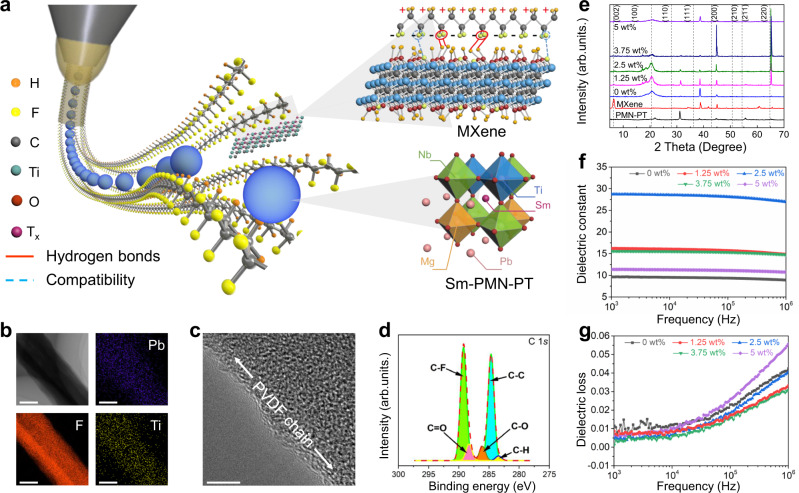
Structure design and characterization of the MXene-enabled piezoelectric composite (MPC) based soft piezoelectric textile (PT). **a** Schematic illustration of electrospinning procedure for MPC textiles synthesis. Inset: Intermolecular interaction between MXene and PVDF; and 2 × 2 × 2 supercell of Sm-PMN-PT as a ceramic filler. **b** Scanning electron microscope (SEM) images and Energy-dispersive spectrometer (EDS) mapping spectra of Pb, F, and Ti elements in the as-prepared composite film. Scale bars: 200 nm. **c** High resolution SEM image of an as-electrospun single fiber. Scale bar: 5 nm. **d** C 1*s* X-ray photoelectron spectrometer (XPS) spectra of as-prepared PT. **e** X-ray diffraction (XRD) spectra of undoped textiles and the prepared MPC textile with varying MXene mass fractions. **f** Frequency-dependent dielectric permittivity of fabricated MPC textile with diverse MXene mass fractions. **g** Frequency-dependent dielectric loss of fabricated MPC textile with diverse MXene mass fractions.

To bring the aforementioned proposed principle and strategy into reality, electrospinning was adopted to construct MXene doped fluoropolymer due to its inherent merit of integrating in-situ stretching with local poling in one step. Figure 2a elucidates the synthesis procedure and composition of the MXene/Sm-PMN-PT/PVDF nonwoven piezoelectric composites, in which a suspension of MXene powder, Sm-PMN-PT particles and PVDF was used as a precursor for subsequent electrospinning (Supplementary Fig. 8). Sm-PMN-PT was selected as the ceramic fillers because of its large piezoelectric coefficient (~1500 pC/N)^2^ in comparison with the conventional piezoelectric ceramics such as BTO (~190 pC/N)^48^, PZT (560 pC/N)^49^ and PMN-PT (~620 pC/N)^3^ (bottom inset of Fig. 2a). Meanwhile, in addition to intermolecular linkage bound with PVDF polymer chain (upper inset of Fig. 2a), the introduction of MXene lamellae efficiently enhances the conductivity and mechanical ductility of the precursor and thus promotes the subsequent local electric poling and in-situ mechanical stretching of polymer composites during electrospinning, which is beneficial to achieving the zigzag alignment of −CH_2_ and −CF_2_ and thus the all-*trans* conformation within PVDF polymer (Supplementary Fig. 9).

Figure 2b reveals the morphology and element structure of the as-synthesized piezoelectric nonwoven textiles consisting of randomly stacked molten PVDF/Sm-PMN-PT/MXene nanofibers through the electrospinning method. The incorporation of MXene nanosheets exhibits a negligible impact on the morphology of the fibrous configuration (Supplementary Fig. 10). Energy-dispersive spectrometer (EDS) mapping spectra of Pb, F, and Ti elements further confirms the uniform distribution of Sm-PMN-PT, PVDF and MXene in the composite films (Fig. 2b and Supplementary Fig. 11). Note that the formation of TiO_2_ originates from the oxidation of MXene on the surface (Supplementary Figs. 12, 13). According to the high-resolution transmission electron microscope images, PVDF polymeric chains aligning along the inorganic-organic interface validate the zigzag alignment of −CH_2_ and −CF_2_ (Fig. 2c). In addition, X-ray photoelectron spectrometer (XPS) spectra (Fig. 2d and Supplementary Fig. 14) evidently demonstrates that the oxygen functionalities and hydrogen bonds were anchored on the surface of the as-synthesized nanofibers, where the emerging peaks of C−O and C=O groups resulting from the MXene incorporation suggest that strong interactions such as hydrogen bonds are formed between the −OH and −O_x_ functional groups in the MXene flakes and the −CF_2_ groups within PVDF, evidencing the aforementioned hypothesis. Higher binding energy peaks observed in O 1*s* region (Supplementary Fig. 15) for C−Ti−O and C−Ti−OH demonstrate that hydroxy termination is dominant on the surface of the flakes.

Figure 2e elucidates the X-ray diffraction (XRD) pattern of the as-synthesized nanofibers and pristine MXene and Sm-PMN-PT powders. The characteristic 2θ peak of the Ti_3_C_2_T_x_ lamellae is 6.48° corresponding to (002) lattice plane with a d-spacing of 13.66 Å, indicative of successful removal of the Al atom layer from the multilayer Ti_3_AlC_2_ MAX phase during the chemical etching process (Supplementary Fig. 16)^50^. Apparently, the peak intensity (002) of the composites is lower than that of the pristine MXene as the addition of PVDF molecules enhances the distance between MXene flakes. Furthermore, six diffraction peaks at 20.5°, 31.4°, 38.5°, 44.7°, 50.4° and 56.1° were ascribed to the crystal planes of (100), (110), (111), (200), (210), and (211), respectively. The existence of these peaks confirms that the Sm-PMN-PT ceramic fillers in the composite film retain polycrystalline perovskite structure^51^. In addition, the typical diffraction peak at 20.5° for the crystal plane of (110) maximizes at the Ti_3_C_2_T_x_ mass fraction of 2.5 wt%, indicating a larger ratio of local all-*trans* conformation (polar β phase) when compared with other versions.

The frequency dependencies of dielectric relative permittivity and dielectric loss of as-prepared MPC textiles and undoped textiles were respectively presented in Fig. 2f, g as a function of MXene inclusion amount at room temperature. The dielectric permittivity for all the samples slightly declines with increasing frequency as a consequence of relaxation (Fig. 2f). It is found that the dielectric permittivity pronouncedly undulates with MXene doping amount and reaches a maximum at 2.5 wt% over a frequency range of 1 kHz–1 MHz, while the dielectric loss maximizes at 5 wt%. This is because the proper inclusion of conductive MXene nanosheets is favorable for interfacial polarization between fillers and polymer matrix, whereas the excessive addition of MXene into polymer composites gives rise to an augmented leaking current and thereby a larger dielectric loss.

### Characterization and phase-field simulation

**Fig. 3:**
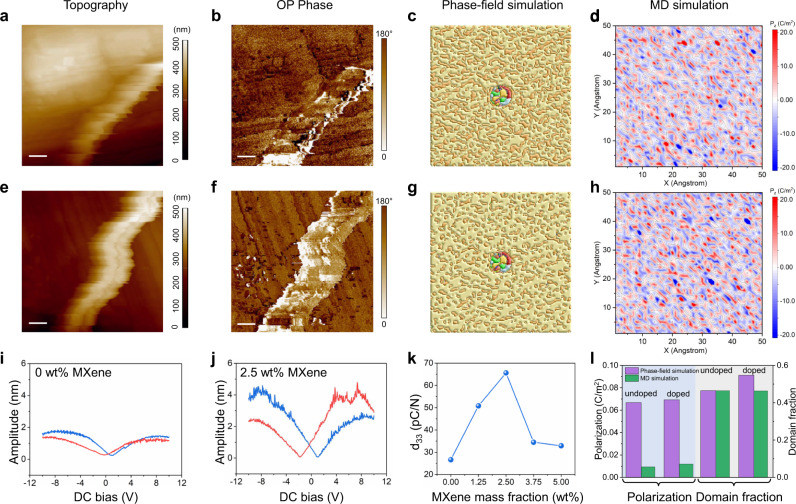
Characterization and simulations for the as-synthesized electrospun fibers. **a**–**d** Piezoelectric force microscope (PFM) Topography (**a**), Out-of-plane (OP) PFM phase image (**b**), phase-field simulation of domain structure (**c**), MD calculation of polarization (**d**) of a single undoped nanofiber. Scale bars: 200 nm. **e**–**h** PFM Topography (**e**), PFM phase image (**f**), phase-field simulation of domain structure (**g**), MD calculation of polarization (**h**) of a single MPC nanofiber. Scale bars: 200 nm. **i**, **j** PFM characterized butterfly amplitude loops for undoped fibers (**i**) and 2.5 wt% MXene doped fibers (**j**) under a direct current (DC) bias from −12 V to 12 V. **k** The PFM measured piezoelectric coefficient (d_33_) of as-prepared nanofibers as a function of MXene mass fractions. **l** Comparison of the phase-field simulation and MD simulation on the spontaneous polarization and out-of-plane domain fractions for the undoped and MXene-doped samples.

To further analyze the interfacial coupling effect between the fluoropolymer matrix and the inclusion, piezoelectric force microscopy (PFM) is employed to assess the ferroelectric and piezoelectric properties of as-electrospun nanofibers with or without MXene doping. The PFM amplitude, PFM phase, phase-field simulated domain structure, and MD calculated polarization were presented for a single undoped Sm-PMN-PT/PVDF fiber (Fig. 3a–d) and a MPC fiber (Fig. 3e–h). The PFM results show a larger area of single ferroelectric domain observed in the MPC fiber in comparison with that in the undoped fiber, implying an enhanced piezoelectric response after MXene addition. This was both certified by the phase-field simulation (Fig. 3c, g) of the ferroelectric domain structure and MD calculation results (Fig. 3d, h) that the out-of-plane domain size of polymer composite becomes larger in the vicinity of the ceramic particle by adding MXene flakes. The phase-field simulation reveals a multi-domain state with 180 degrees domains in the polymer matrix for both undoped and MPC fibers, where the MPC fiber possesses a larger domain fraction with polarization aligned along the poling direction (PD) of electrospinning compared with that of the undoped fiber. Moreover, the embedded Sm-PMN-PT particle maintains a polycrystalline structure as its coercive field is much higher than the in-situ poling field (1.2 kV/cm) of electrospinning^2^.

The modulated dipole structure of MPC fibers with increased polarization further gives rise to an increased piezoelectric response. PFM measurements show that all the as-synthesized electrospun fibers exhibit a butterfly-shape hysteresis of the amplitude versus bias, where the MPC fiber displays a higher amplitude (Fig. 3j) than the undoped one (Fig. 3i), implying a larger piezoelectric coefficient (d_33_). As displayed in Fig. 3k, the d_33_ derived from PFM test first increases and then decreases upon increasing the MXene doping amount, with a maximum d_33_ achieved at 2.5 wt% of MXene. It is worth noting that both phase-field simulation and MD calculation follow a similar changing tendency of polarization as well as out-of-plane domain proportion to the PFM results (Fig. 3l), which verifies the accuracy and reliability of our proposed theoretical modeling.

**Fig. 4:**
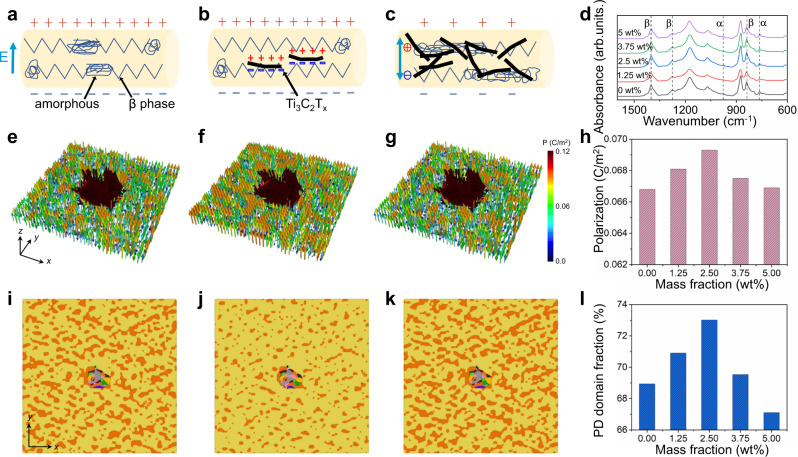
Mechanism of as-electrospun nanofibers via doping MXene nanosheets. **a**–**c** Schematic of the polarization process of electrospun nanofibers doped with **a** no MXene, (**b**) proper amount of MXene, and **c** excessive amount of MXene. **d** Fourier transform infrared (FTIR) spectrum of the undoped textiles and the prepared MPC textiles with varying MXene mass fractions. **e**–**g** Simulated polarization field in the vicinity of Sm-PMN-PT particle in the nanocomposites doped with **e** no MXene, (**f**) proper amount of MXene, and **g** excessive amount of MXene. **h** Calculated polarization of the MPC textiles doped with various MXene amount. **i**–**k** Simulated domain structure in the vicinity of Sm-PMN-PT particle in the nanocomposites doped with **i** no MXene, (**j**) proper amount of MXene, and **k** excessive amount of MXene. **l** Calculated poling direction (PD) domain fraction of the prepared textiles as a function of MXene doping amount.

To achieve a more intuitive understanding of the functionality and mechanism of MXene lamellae addition, a schematic was utilized to elaborate the β crystalline phase conformation and orientation during the electrospinning of the MXene/Sm-PMN-PT/PVDF composites. For bare PVDF nanofibers (Fig. 4a), β phase crystalline can be partially aligned by the in-situ poling while some localized amorphous microstructures still exist. The situation was changed once a small amount of MXene (1.25 wt% and 2.5 wt% in our case) is added into the PVDF-based composite, as shown in Fig. 4b. In addition to the hydrogen bonds formed between PVDF chains and MXene sheets, the applied electric field yields inductive charges on the surface of conductive MXene nanosheets and thereby provokes a greater Coulomb force during the electrospinning. This electrostatic force further attracts PVDF chains to crystallize on the MXenes surface in the zigzag arrangement, which expedites the transformation of local amorphous regions into the β crystalline phase. Consequently, the β phase is enhanced when compared with pure PVDF nanofibers (Fig. 4a). However, superfluous addition leads to a stack of MXene nanosheets and suppresses the orientation and alignment of polymeric chains in the way of forming β phase crystalline. In the meantime, the connection of these stacked MXene flakes boosts the leaking current, which hinders the effective dielectric permittivity (ε_33_) and piezoelectric coefficient (d_33_) of the composite. To verify this proposed modeling on the crystallinity and phase shift, the Fourier transform infrared (FTIR) spectrum of the undoped textiles and MPC textiles doped with various MXene mass fractions were revealed in Fig. 4d. The adsorption peaks at 763 cm^−1^ and 973 cm^−1^ are attributed to α-phase crystalline structure of PVDF and the adsorption peak at 841 cm^−1^, 1276 cm^−1^ and 1429 cm^−1^ are ascribed to a β-phase crystalline structure^52^. According to Beer-Lambert equation^5^, the fraction of the β phase (F_β_) of as-synthesized nanofibers can be attained as a function of MXene loading amount (Supplementary Fig. 17), where the highest β phase of 83.4% occurs at the loading of 2.5 wt% MXene. Consequently, the phase evolution trend upon MXene doping revealed by the FTIR test agrees well with the proposed principle.

To further testify the above hypothesis and shine some light on the mechanism, phase-field simulation was carried out to numerically illustrate the impact of conductive fillers on the domain structure and polarization of the ferroelectric composites. As shown in Fig. 4e–g, incorporation of a proper content (2.5 wt%) of MXene lamellae notably improves the polarization of the as-electrospun composite, whereas further accretion jeopardizes the polarization and may even cause it to fall below the undoped counterpart (Fig. 4h), which is in good accordance with foregoing deduction and FTIR test. Furthermore, as for the simulated domain structure (Fig. 4i–k), the incorporation of MXene drives the polarization toward the out-of-plane direction parallel to the electrospinning poling field when compared with the undoped one, which is also consistent with the PFM results. A loading of 2.5 wt% MXene contributes to an enhancement of the volume fraction of PD out-of-plane domain from 67.8 to 72.6% (Fig. 4l and Supplementary Figs. 18, 19). It is believed that the net out-of-plane domain is responsible for the augmented β phase content and corresponding larger piezoelectric response. Given that the piezoelectric coefficient is proportional to spontaneous polarization and permittivity (*d*_*33*_ = 2*P*_*s*_*ε*_*33*_*Q*_*33*_), a proper doping of the conductive component is proven to be an efficient approach to upgrade the piezoelectric performance of polymer composites.

**Fig. 5:**
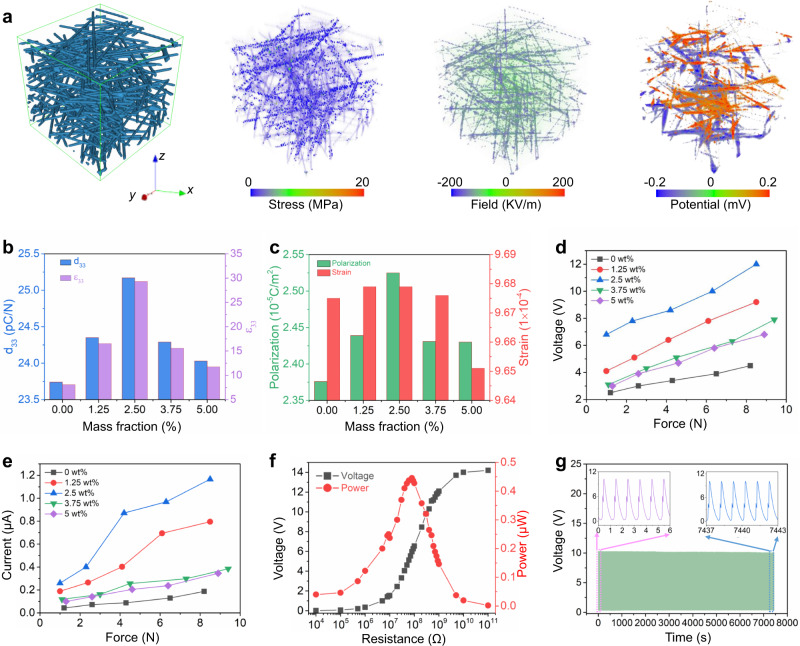
Characterization and phase-field simulation results of the as-electrospun textiles. **a** The visualized structural modeling, stress distribution, electric field distribution, and piezoelectric potential distribution of as-prepared MPC textiles. Note: The applied stress is fixed at 10^6^ Pa along the *z*-axis for the simulation. **b** The calculated piezoelectric coefficient (d_33_) and dielectric constant (ε_33_) of the as-electrospun textiles doped with various MXene mass fractions. **c** The calculated average polarization and strain of the as-electrospun textiles doped with various MXene mass fractions. **d**, **e** Output voltage (**d**) and current (**e**) of the as-electrospun textiles as a function of applied mechanical force. **f** Impedance response of prepared 2.5 wt% MXene doped MPC. **g** Durability of the as-prepared MPC.

To understand the role of MXene doping on the percolation effect in the as-electrospun composites nanofibers, a phase-field simulation of effective properties was implemented to analyze the dependence of the MXene doping amount on the dielectric, piezoelectric and mechanical properties of the electrospun unmodified and MPC textiles. As displayed in Fig. 5a, a three-dimensional fibrous configuration was randomly computer-generated to imitate the structure of electrospun nonwoven textiles (Supplementary Fig. 10). The stress distribution, electric field and electric potential of MPC textiles under constant applied stress of 1 × 10^6^ Pa along the out-of-plane (z) direction are calculated. As displayed in Fig. 5b, the calculated piezoelectric coefficient d_33_ and dielectric permittivity ε_33_ undulate with increasing MXene mass fraction from 0 to 5.0 wt%, reaching a maximum value at 2.5 wt%. A similar trend is observed on both the d_33_ meter measured d_33_ value and the calculated strain-induced piezoelectric polarization, two of which fluctuate with the loading of MXene flakes and reaches a maximum at 2.5 wt% (Fig. 5c and Supplementary Fig. 20). On the other hand, the stiffness coefficient retains almost the same at low MXene doping amount but remarkably decreases at high MXene doping level, indicating a hardening of the composites with high contents of doped MXene flakes.

### MPC textiles for energy harvesting and sensing

To explore the device performance of the fabricated MPC textiles in response to external stimulation, the output piezoelectric signals were systematically characterized under a diversity of applied forces varying from 1 to 9 N, as plotted in Fig. 5d, e. Notably, the electric outputs of the MPC textiles are proportional to applied stress and the MXene doped devices deliver larger voltage and current than the unmodified version. Furthermore, under a fixed external force of 3 N, the signal intensity varies with the changing doping amount of MXene and the 2.5 wt% MXene doped nanocomposite yields a gain of 160% when compared with the undoped sample (Supplementary Figs. 21, 22). It is worth pointing out that the electric outputs follow an analogous changing tendency as the calculated piezoelectric coefficient upon conductive filler doping (Fig. 5e), which convincingly confirmed the validation and feasibility of our proposed theoretical modeling and corresponding calculation. Consequently, all the following measurements were carried out using an optimal MXene mass fraction of 2.5 vol% because of its favorable piezoelectric properties. Figure 5f shows the impedance response of the prepared MPC textile doped with 2.5 wt% MXene under various resistances from 10 KΩ to 100 GΩ, where the maximum output power was achieved at 80 MΩ. The mechanical stretchability and robustness of as-synthesized MPC textiles were testified by the long-term fatigue test as illustrated in Fig. 5g, where the output voltage retains a constant value when subjected to more than 7000 cycles of 5 N impact force application, demonstrating an excellent mechanical robustness and reliability for practical scenarios.

**Fig. 6:**
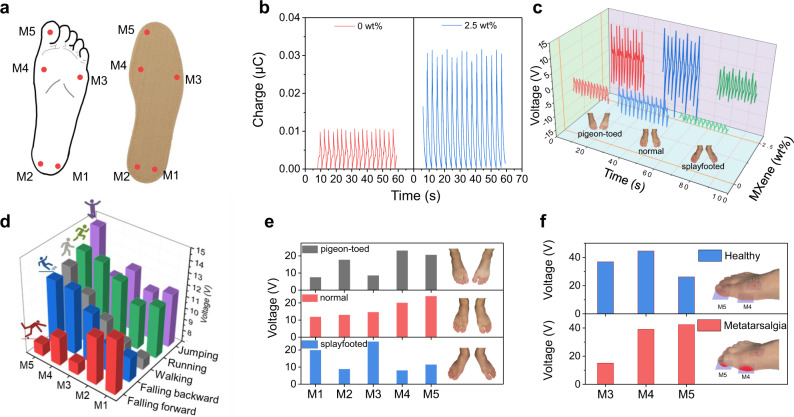
Output performance and sensing application of as-prepared MPC based soft PT sensors. **a** Schematic of a smart insole integrated with five PT sensors on five different sites to form a foot sensor network. **b** Piezoelectric charges of undoped fiber and MPC fiber on M5 site in response to an adult’s (75 kg) toe pressing. **c** Dynamic output voltages of undoped fiber and MPC fiber on M5 site when subjected to normal, pigeon-toed and splayfooted postures. **d**–**f** Signals profiles for gait monitoring (**d**), posture habits recognition (**e**), and Metatarsalgia prognosis (**f**).

To verify the sensing capability of MPC textile for active biomonitoring, a self-powered gait monitoring system was developed. As schemed in Fig. 6a, by attaching as-synthesized devices onto five different positions (M1–M5) of a conventional insole (see Supplementary Note 1, Supplementary Figs. 23, 24 for details), the stress distribution of the foot can be spontaneously detected. It can be clearly seen that the incorporation of MXene triggers a much larger amplitude of output charge than the undoped one when the big toe pressing the device on site of M5 (Fig. 6b) regardless of walking postures including pigeon-toed, normal and splayfooted (Fig. 6c). Furthermore, various gait patterns of walking, running, jumping, falling forward, and falling backward can be accurately recognized and distinguished with regards to the dynamic signal mapping among the five units (Fig. 6d and Supplementary Movie 1). It is interesting that the falling forward and falling backward possess adverse signal mapping profiles due to the opposite moving direction of the falling body. Meanwhile, the signal profile of walking, running, and jumping acquired by the five positions gradually rises up as a result of augmented stamped force exerted on the insole. Aside from the real-time pressure distribution recognition, the dynamic stride frequency and amplitude can be efficiently identified and recorded by the interval and amplitude of the signal waveforms, respectively (Supplementary Fig. 25). Note that even though the as-electrospun PTs might deliver smaller output signals than the triboelectric devices^53^, they do not need an extra mobile component like triboelectric devices to trigger electricity generation, allowing for all-in-one configuration and biocompatibility^12^. To demonstrate the potential of MPC textiles in identifying walking habits, the posture abnormity like pigeon-toed or splayfooted was further distinguished using the as-prepared smart insole (Fig. 6e and Supplementary Fig. 26). Apparently, the splayfoot renders a much higher pressure on the lateral side but lower pressure on the inner side of insole in comparison with the normal posture, which gives rise to a larger signal amplitude on position M1 and M3. In contrast, the pigeon-toed walking posture triggers a much higher pressure on the inner side but lower pressure on the lateral side of the insole when compared with the normal posture, leading to a larger amplitude on position M2, M4 and M5. Aside from the gait monitoring, the prepared smart insole based on MPC textiles also reveals capability in clinic diagnosis. As a widespread foot condition, Metatarsalgia brings about a shape deformation of the metatarsal region and subsequent sharp pain in the ball of the foot. Figure 6f unveils and compares the walking signal profiles of a healthy person and  a Metatarsalgia patient on the metatarsal region (M3, M4, M5). Due to the classical symptom of the curled toe, a signal contour with larger intensity on M5 and smaller intensity on M3 was recognized for the Metatarsalgia patient when compared to that of the healthy tester (Supplementary Fig. 27), which demonstrates the capacity of MPC textiles in clinic prognosis of Metatarsalgia.

## Discussion

A high-performance piezoelectric composite was fabricated via termination engineering using Ti_3_C_2_T_x_ MXene templating. To understand the interfacial coupling effect of the piezoelectric composites, PFM characterization in combination with phase-field simulation and MD calculation have been utilized to conduct a comprehensive analysis of the functionality and mechanism of MXene inclusion into ferroelectric polymer composites. A strong hydrogen bonding between the hydroxyl terminations on MXene nanosheets and −CF_2_ moieties in PVDF chains is responsible for the long-range growth of TTTT configuration and alignment of domain orientation, which efficiently boosts the β phase content and piezoelectric coefficient. We then translated the hydrogen bond anchoring strategy into reality by synthesizing the Ti_3_C_2_T_x_/Sm-PMN-PT/PVDF composites-based soft PTs via electrospinning. It is found that the incorporation of 2.5 wt% Ti_3_C_2_T_x_ into Sm-PMN-PT/PVDF composites leads to an output gain of 160% when compared with the undoped version. Furthermore, the as-electrospun MPC could be easily integrated into the conventional insole for active gait patterns monitoring, walking habits identification and Metatarsalgi prognosis. This work theoretically and experimentally looks into the underlying mechanism of the interfacial coupling effect of piezoelectric nanocomposites, opening up a paradigm for the development of high-performance wearable electronics.

## Methods

### Synthesis of Sm-PMN-PT piezoelectric ceramic particles

The composition of the Sm-PMN-PT is Pb_0.97_Sm_0.02_[(Mg_1/3_Nb_2/3_)_0.7_Ti_0.3_]O_3_^3^. MgNb_2_O_6_ powder was initially prepared using a solid-state reaction at 1200 °C. Pb_3_O_4_, MgNb_2_O_6_, TiO_2_, and Sm_2_O_3_ powders were mixed by ball-milling overnight as wet dispersion. Calcination of the mixed powders was conducted in a non-contaminated furnace at 850 °C for 2 h.

### Synthesis of layered MXene nanosheets

Firstly, 10 ml ultrapure water was utilized to dilute 30 ml 12 M hydrochloric acid solution (Chron chemicals Co., Ltd, China) to obtain 40 ml 9 M HCl. Subsequently, 2 g LiF (Aladdin, China) was added into 40 ml 9M HCl followed by magnetic stirring for 5 min in order to obtain a hydrofluoric acid solution. Next, 1.2 g Ti_3_AlC_2_ powder (MAX phase, purchased from 11 Technology Co., Ltd., China) was weighed and slowly added to the mixed solution for water bath stirring under a rotation speed of 400 rpm for 24 h at 35 °C. Then, the reaction product was centrifugated at 1737 × *g* for 5 min several times and rinsed with deionized (DI) water until the pH value reached 6. After suction filtration and vacuum drying (45 °C, 24 h) followed by an ice bath ultrasonic, the MXene nanosheets were attained for spare.

### Fabrication of MPC textile-based sensor

First, the as-synthesized MXene powders of 0, 29.5, 59, 88.5, 118 mg, corresponding to the mass fraction of 0, 1.25, 2.5, 3.75, 5 wt% respectively, were dispersed in a beaker of 6 mL dimethylformamide (DMF) and 4 mL acetone mixed solvent and ultrasonically treated for 20 min to evenly disperse nanosheets in DMF solvent. Then, 118 mg Sm-PMN-PT nanoparticles together with 2.25 g PVDF powder were added into the mixed solution via magnetic stirring at 50 °C for 2.5 h in a water bath to obtain a stable and uniform electrospinning precursor solution. After solution agitation, ultrasonic treatment for 30 min allows nanoparticles to disperse evenly. Subsequently, the as-received solution was added into a 10 mL plastic syringe with a needle orifice of 25 gauge for electrospinning. The electrospinning was carried out for 2 h with a gap distance of 15 cm, a pushing speed of 0.6 mL/h and an electric field of 1.2 KV/cm at room temperature and 42% humidity (Zhiyan Technology Co., Sichuan). Then, as-electrospun textile was adhered by two pieces of aluminum tapes as electrodes and tailored into small pieces with a size of 3.0 cm × 3.0 cm. Finally, the whole device was packaged using PET substrates and transparent medical tape to build up a MPC textile-based sensor.

### Characterization and measurement

The *d*_33_ values were characterized using a ZJ quasi-static *d*_33_ meter developed by the Chinese Academy of Sciences with a frequency of 110 Hz and a mechanical load of 0.25 N. A Keithley 6514 electrometer system was used to collect the output signals of the prepared devices. The dielectric properties of the electrospun films were measured using an Agilent 4294A precision impedance analyzer with a film thickness of 15 µm and an area of 1 cm × 1 cm. A field emission scanning electron microscopy (FESEM, Apero, Thermo, U.S.A.) was employed for morphology characterization. Piezoresponse force microscopy (PFM, NT-MDT Spectrum Instruments) was used to image the ferroelectric domain structures using a contact lithography mode with a tip curvature radius of 35 nm and a tip height of 14–16 µm, where an alternating-current voltage is applied to the metallic disk (=150 kHz, U_RMS_ = 0.5 V) and two Stanford Research 830R lock-in amplifiers are used to monitor the amplitude and phase of the deflection and torsion of the cantilever. Conductive Platinum-Iridium silicon cantilevers (NSG01/Pt) were used for the PFM characterization with detailed parameters listed in Supplementary Table 1. The phase purity and crystal properties of the samples were analyzed by an X-ray diffractometer (XRD: D8 Advance, Bruker-AXS, Germany) equipped with Cu-Kα radiation (λ = 1.5418 Å). An XPS (ESCALAB 250Xi) was used with an Al Kα excitation (1486.8 eV). The chemical composition and valence states were characterized by X-ray photoelectron spectroscopy (XPS, model ESCALAB 250, Al Kα, hυ = 1486.6 eV). The FTIR spectra were performed on Fourier infrared spectrometer (Smartlab 9, Rigaku, Japan). A linear motor with tunable frequency, amplitude, velocity and force was adopted to apply a diversity of stress and strain on the samples.

### Phase-field simulation of the ferroelectric domain structures

In the phase-field simulation, the time-dependent Ginzburg-Landau equation was employed to describe the evolution of the polarization^54^ [Eq. 1],

1 $$\frac{\partial {P}_{i}({{{{{\boldsymbol{r}}}}}},t)}{\partial t}=-L\frac{\delta F}{\delta {P}_{i}\left({{{{{\boldsymbol{r}}}}}},t\right)},\; ({{{{{\rm{i}}}}}}=1,2,3)$$

where *L* is the kinetic coefficient, *t* is the time, *F* is the total free energy of the system, ***r*** = (*x*, *y*, *z*) is the spatial position vector, and _*Pi*_ (***r****, t*) is the polarization field. *δF ∕ δ*_*Pi*_(***r****, t*) is the thermodynamic driving force for the spatial and temporal evolution of polarization.

The total free energy of the system includes contributions from the bulk free energy *f*_*bulk*_, the elastic energy *f*_*elastic*_, the electrostatic energy *f*_*electric*_, and the gradient energy *f*_*grad*_: [Eq. 2]

2 $$F=\int\limits_{V}\left[{f}_{{bulk}}+{f}_{{elastic}}+{f}_{{electric}}+{f}_{{grad}}\right]dV$$

In particular, the bulk free energy density *f*_*bulk*_ is expressed following the Landau theory. The bulk free energy density of Sm-PMN-PT is written as a 6-th order polynomial^55^

3 $${f}_{{bulk}}=	 {{{{{{\rm{\alpha }}}}}}}_{1}\left({P}_{x}^{2}+{P}_{y}^{2}+{P}_{z}^{2}\right)+{{{{{{\rm{\alpha }}}}}}}_{11}\left({P}_{x}^{4}+{P}_{y}^{4}+{P}_{z}^{4}\right)+{{{{{{\rm{\alpha }}}}}}}_{12}\left({P}_{x}^{2}{P}_{y}^{2}+{{P}_{x}^{2}P}_{z}^{2}+{{P}_{y}^{2}P}_{z}^{2}\right)\\ 	 {+{{{{{\rm{\alpha }}}}}}}_{111}\left({P}_{x}^{6}+{P}_{y}^{6}+{P}_{z}^{6}\right)+{{{{{{\rm{\alpha }}}}}}}_{112}\left({P}_{x}^{2}({P}_{y}^{4}+{P}_{z}^{4})+{P}_{y}^{2}({P}_{z}^{4}+{P}_{x}^{4})+{P}_{z}^{2}({P}_{x}^{4}+{P}_{y}^{4})\right)\\ 	 {+{{{{{\rm{\alpha }}}}}}}_{123}{P}_{x}^{2}{P}_{y}^{2}{P}_{z}^{2}$$

where *α*_*i*_, *α*_*ij*_, *α*_*ijk*_ are the Landau coefficients [Eq. 3]. The bulk free energy density of PVDF is expressed as [Eq. 4]

4 $${f}_{{bulk}}={{{{{{\rm{\alpha }}}}}}}_{1}{P}_{x}^{2}+{{{{{{\rm{\alpha }}}}}}}_{2}{P}_{y}^{2}+{{{{{{\rm{\alpha }}}}}}}_{3}{P}_{z}^{2}+{{{{{{\rm{\alpha }}}}}}}_{33}{P}_{z}^{4}+{{{{{{\rm{\alpha }}}}}}}_{333}{P}_{z}^{6}$$

The expression is established following a uniaxial anisotropy with spontaneous polarization along the *z* axis (which is the PD during electrospinning), while polarization components along *x* and *y* axes assume paraelectric properties with higher order terms neglected.

Incorporation of MXene was considered through treating different background dielectric constants **ε**_**b**_ by solving the electrostatic equilibrium equation^56^,

5 $$\nabla \cdot \left({\varepsilon }_{0}{{{{{{\boldsymbol{\varepsilon }}}}}}}_{b}{{{{{\bf{E}}}}}}+{{{{{\bf{P}}}}}}\right)=0$$

where **ε**_***b***_ correlated to MXene doping amount (Fig. 2f) to account for the variation of β phase fraction [Eq. 5].

The evolution of the domain structure was simulated for a single electrospun fiber under an applied electric field of 1.2 × 10^5^ V/m, following the in-situ poling field (1.2 KV/cm) of electrospinning. The simulations were performed in a three-dimensional system with a total size of 512 nm × 512 nm × 512 nm containing a single Sm-PMN-PT particle (diameter = 100 nm) in a PVDF matrix, which was then discretized into a three-dimensional array of 128 × 128 × 128 grids with a grid size of Δx = Δy = Δz = 4 nm. Periodic boundary conditions were employed for the polarization field, the electric field, and the mechanical displacement field. The material constants of Sm-PMN-PT and PVDF are listed in Supplementary Tables 2 and 3, respectively. All phase-field simulations in the present work were performed using the MuPRO Ferroelectric module for linux cluster software released from Mu-PRO LLC.

### Effective properties calculation of the piezoelectric composites

These simulations were performed using Mu-PRO Effective Properties module for linux cluster released from Mu-PRO LLC to systematically study and compare the stress transfer capability and electromechanical coupling effect of the electrospun nanofiber architecture with and without the MXene incorporation. The composite system consists of randomly oriented Sm-PMN-PT filler fibers with a diameter d = 200 nm at a total content of 3.23 vol% in a PVDF matrix. The total system size was taken as 12.8 µm × 12.8 µm × 12.8 µm, which was discretized into a three-dimensional array of 128 × 128 × 128 grids. A three-dimensional periodic boundary condition was employed.

The effective dielectric constant ${{{{{{\boldsymbol{\varepsilon }}}}}}}_{r}^{{eff}}$ and piezoelectric coefficient ${{{{{{\bf{d}}}}}}}_{r}^{{eff}}$ of the composite system was calculated by modeling the coupled response of the electric displacement field **D**(***r***) and the strain **ε**(***r***) of the composites to a small applied testing electric field **E**_app_ under a stress-free condition. The material response was simulated by solving the electrostatic and elastic equilibrium equations coupled with linear constitutive relations^5^, i.e.,

6 $$\nabla \cdot {{{{{\bf{D}}}}}}=\nabla \cdot \left({\varepsilon }_{0}{{{{{{\boldsymbol{\varepsilon }}}}}}}_{r}{{{{{\bf{E}}}}}}+{{{{{\bf{d}}}}}}{{{{{\boldsymbol{\sigma }}}}}}\right)=0$$

7 $$\nabla \cdot {{{{{\boldsymbol{\sigma }}}}}}=\nabla \cdot \left({{{{{\bf{c}}}}}}{{{{{\boldsymbol{\varepsilon }}}}}}-{{{{{{\bf{cd}}}}}}}^{T}{{{{{\bf{E}}}}}}\right)=0$$

where *ε*_0_ is the vacuum permittivity, **ε**_*r*_(***r***) is the relative dielectric constant of the local phase, **c**(***r***) is the elastic stiffness, and **d**(***r***) is the piezoelectric coefficient [Eqs. 6, 7]. A Fourier-spectral iterative-perturbation method^57^ is employed for obtaining the numerical solution. The effective material properties of the composites were then calculated following ${\varepsilon }_{0}{{{{{{\boldsymbol{\varepsilon }}}}}}}_{r}^{{eff}}{{{{{{\bf{E}}}}}}}_{{{{{{\rm{app}}}}}}}=\left\langle \Delta {{{{{\bf{D}}}}}}\right\rangle$ and ${{{{{{{\bf{d}}}}}}}_{r}^{{eff}}}^{T}{{{{{{\bf{E}}}}}}}_{{{{{{\rm{app}}}}}}}=\left\langle \Delta {{{{{\boldsymbol{\varepsilon }}}}}}\right\rangle$, where $\left\langle \Delta {{{{{\bf{D}}}}}}\right\rangle$ and $\left\langle \Delta {{{{{\boldsymbol{\varepsilon }}}}}}\right\rangle$ are the spatial average of the displacement response and the strain response with the whole system, respectively. The materials constants of the Sm-PMN-PT fillers and PVDF matrix are listed in Supplementary Note 2.

### Molecular dynamics (MD) calculation

In the MD simulations, 60 PVDF molecules with each 30 VDF monomers were constructed at 1 nm from the Ti_3_C_2_T_x_ nanosheets having an initial film density of 1.3 g/cm^3^. The periodic lattice of Ti_3_C_2_T_x_ nanosheets with OH or O surface terminations was directly taken from the literature^58^ and was fixed at their lattice positions, interacting with the PVDF chains only via van der Waals and electrostatic interactions. The Universal Force Field (UFF)^59^ was utilized to describe the intra- and intermolecular interactions for PVDF chains as well as the interaction between the PVDF and Ti_3_C_2_T_x_ nanosheets. The partial charges of the atoms of PVDF chains were assigned using the bond increments method, whereas the charges for the atoms of the Ti_3_C_2_T_x_ substrate were adopted from the first-principle calculations^60^. For equilibration, the PVDF- Ti_3_C_2_T_x_ composite model was initially energy minimized with the energy and force convergences of 1 × 10^−4^ kcal mol^−1^ and 1 × 10^−6^ kcal mol^−1^Å^−1^, and then followed by a 2.5 ns run in the NVT ensemble. After that, an electrical field of 1.0 V/nm was applied to the PVDF chains normal and opposite to the Ti_3_C_2_T_x_ substrate (i.e., *z* direction) for another 2.0 ns, to investigate the polarization performance of the PVDF. The induced polarization was then calculated by summing the dipole moments of all the atoms of the PVDF film within the film volume *V* as [Eq. 8]

8 $${{{{{\bf{P}}}}}}=\frac{1}{V}{\sum }_{i}{{{{{{\bf{u}}}}}}}_{i}=\frac{1}{V}{\sum }_{i}\;{q}_{i}\;{{{{{{\bf{r}}}}}}}_{i}$$

where ${q}_{i}$ and ${{{{{{\bf{r}}}}}}}_{i}$ are the particle charge and Cartesian coordinate vector of the *i*th atom of the PVDF. In addition, the interaction strength between one single PVDF chain and the Ti_3_C_2_T_x_ substrate was estimated by applying a constant force along the *z* direction to each atom of the PVDF chain. A series of MD simulations were performed with varying forces using an incremental step of 0.01 or 0.002 kcal mol^−1^Å^−1^ to produce the minimum pulling force required for fully desorbing the PVDF chain away from the Ti_3_C_2_T_x_ substrate. For all the simulations, the temperature was maintained at 300 K using the Nośe-Hoover thermostat with a relaxation time of 1.0 ps. Newton’s equations of motion were integrated using the velocity-Verlet algorithm with a time step of 1.0 fs and periodic boundary conditions were applied in all three dimensions. The non-bonded van der Waals interactions were cut off at 10 Å and the long-range electrostatic interactions were calculated using the particle-particle particle-mesh solver^61^ with 5 × 10^−6^ accuracy tolerance. All the MD simulations were carried out using the Large-scale Atomic/Molecular Massively Parallel Simulator software^62^.

## Supplementary information

Supplementary Information

Description of Additional Supplementary Files

Supplementary Movie 1

## Data Availability

The data that supports the findings of this study are available from the authors on reasonable request.

## Code availability

The code that supports the findings of this study is available from the authors on reasonable request.
