# Supplementary material for: High-performance piezoelectric composites via β phase programming
Source: Nat Commun. 2022 Aug 18;13:4867. doi: 10.1038/s41467-022-32518-3 (PMC9388583; doi:10.1038/s41467-022-32518-3)
Supplement: Supplementary file 1 — Supplementary Information [file 41467_2022_32518_MOESM1_ESM.docx]

**Supplementary Information**

**High-Performance Piezoelectric Composites via *β* Phase Programming**

Yuanjie Su^†,1^, Weixiong Li^†,1^, Xiaoxing Cheng^†,2^, Yihao Zhou^†,3^, Shuai Yang^4^, Xu Zhang^5^, Chunxu Chen^1^, Tiannan Yang^*,2^, Hong Pan^1^, Guangzhong Xie^1^, Guorui Chen^3^, Xun Zhao^3^, Xiao Xiao^3^, Bei Li^*,5^, Huiling Tai^1^, Yadong Jiang^1^, Long-Qing Chen^2^, Fei Li^*,4^, Jun Chen^*,3^

^1^ State Key Laboratory of Electronic Thin Films and Integrated Devices, School of Optoelectronic Science and Engineering, University of Electronic Science and Technology of China, Chengdu 610054, China

^2^ Department of Materials Science and Engineering, The Pennsylvania State University, State College 16802, USA

^3^ Department of Bioengineering, University of California, Los Angeles, Los Angeles, CA 90095, USA

^4^ Electronic Materials Research Lab, Key Lab of Education Ministry/International Center for Dielectric Research, School of Electronic and Information Engineering, State Key Laboratory for Mechanical Behavior of Materials, Xi’an Jiaotong University, Xi’an 710049, China.

^5^ School of Materials Science and Engineering, Research Center for Materials Genome Engineering, Wuhan University of Technology, Wuhan 430070, China

^†^These authors contributed equally to the work.

^*^Correspondence to: [tuy123@psu.edu](mailto:tuy123@psu.edu) (T. Y.), libei@whut.edu.cn (B. L.), [ful5@xjtu.edu.cn](mailto:ful5@xjtu.edu.cn) (F. L.), jun.chen@ucla.edu (J. C.)

**Keywords**: piezocomposite, interfacial coupling, phase-field simulation, molecular dynamics, wearable bioelectronics


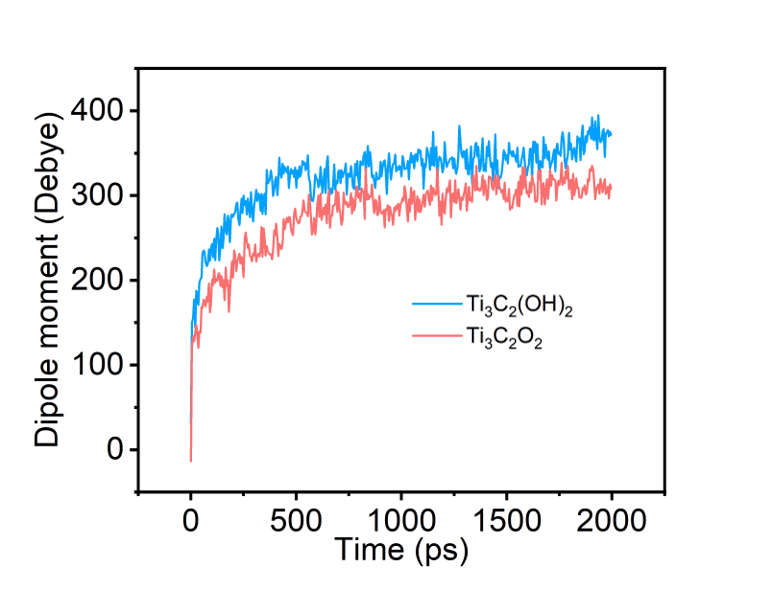


**Supplementary Fig. 1** Molecular dynamics calculated dipole moment evolution of the PVDF chains anchored on Ti_3_C_2_(OH)_2_ and Ti_3_C_2_O_2_ nanosheets under electrical polling.


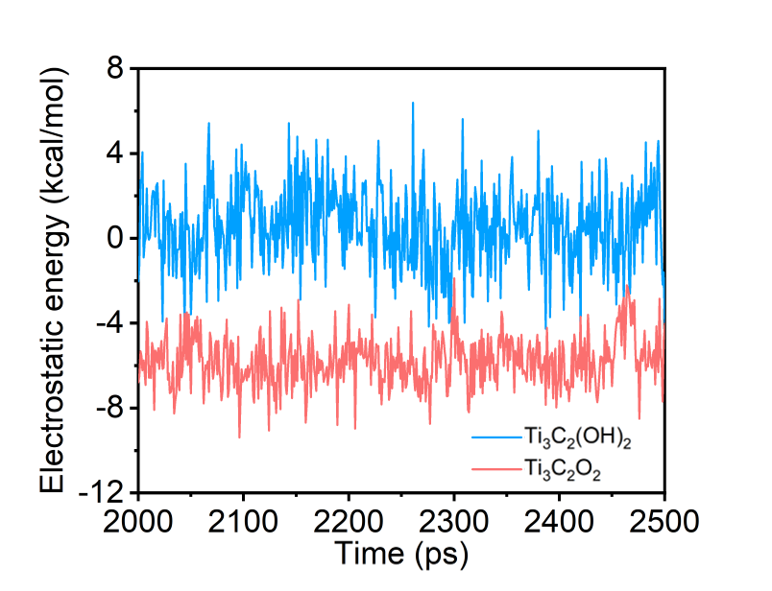


**Supplementary Fig. 2** Molecular dynamics calculated electrostatic energy between the PVDF chains and Ti_3_C_2_T_x_ nanosheets during equilibration.


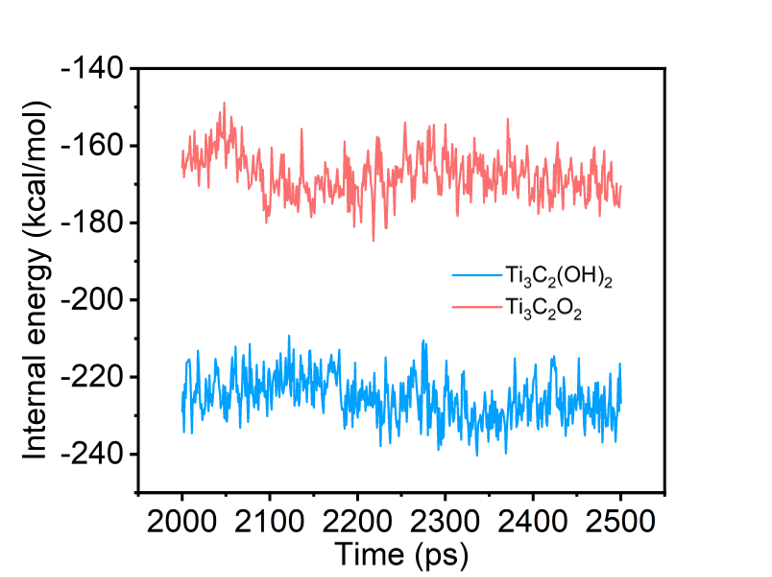


**Supplementary Fig. 3** Molecular dynamics calculated internal energy between the PVDF chains and Ti_3_C_2_T_x_ nanosheets during equilibration.


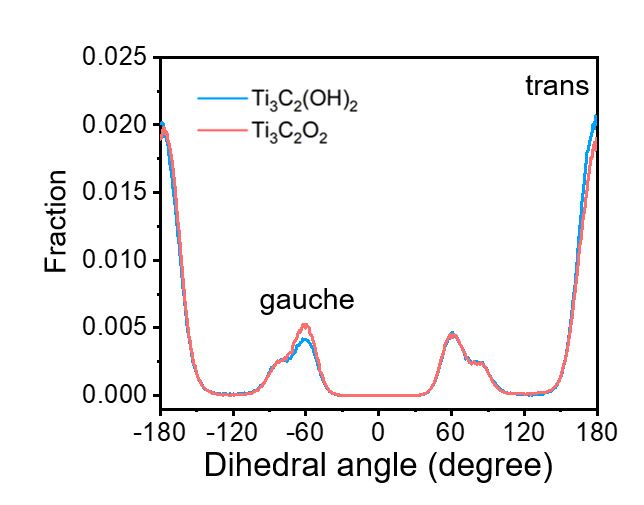


**Supplementary Fig. 4** Molecular dynamics calculated fraction of dihedral angles in bond conformation within the PVDF chains anchored on Ti_3_C_2_(OH)_2_ and Ti_3_C_2_O_2_ nanosheets.

**
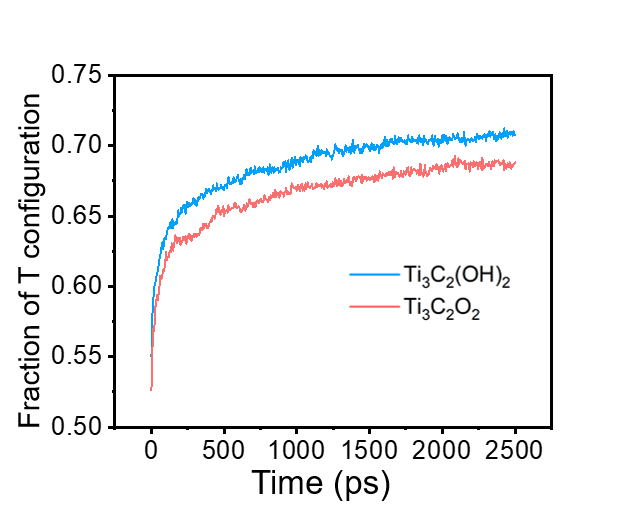
**

**Supplementary Fig. 5** Molecular dynamics calculated fraction of T conformation of the PVDF chains during equilibration.


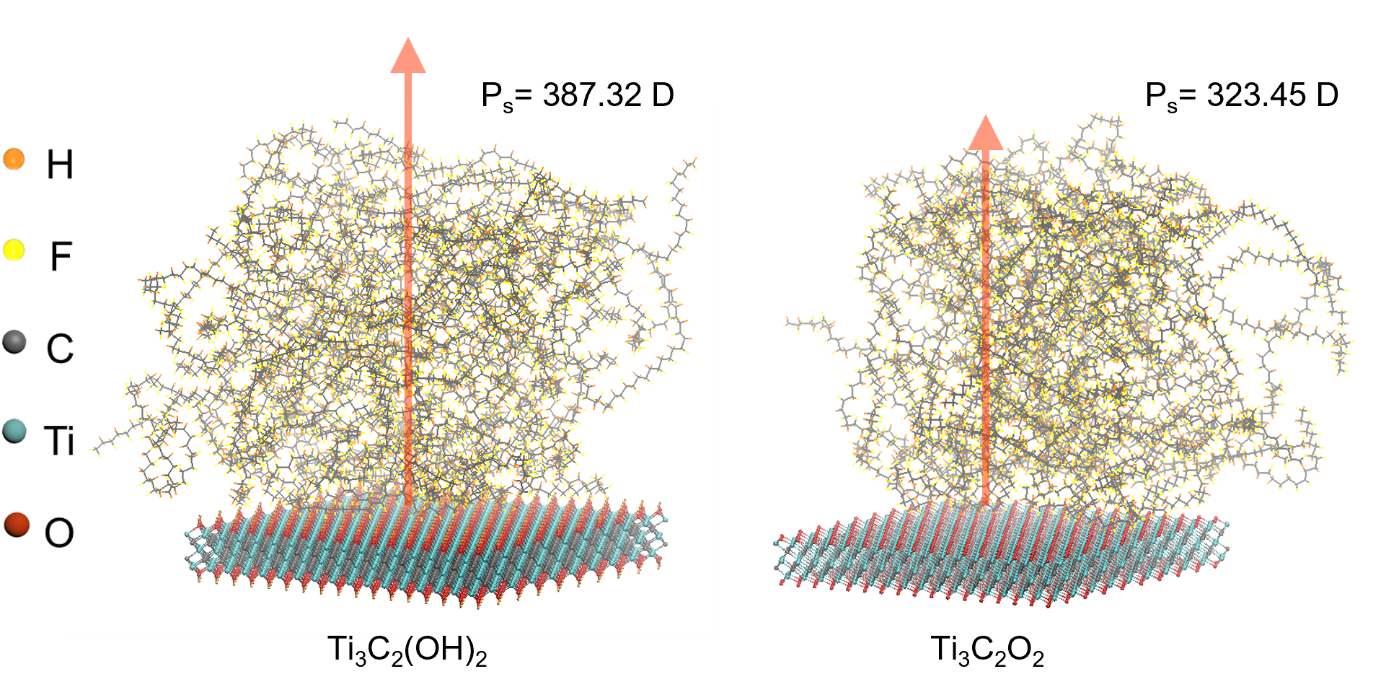


**Supplementary Fig. 6** Final snapshots for Molecular dynamics simulations of the polarization of P(VDF-TrFE) copolymer film on the Ti_3_C_2_(OH)_2_ flakes and Ti_3_C_2_O_2_ flakes.


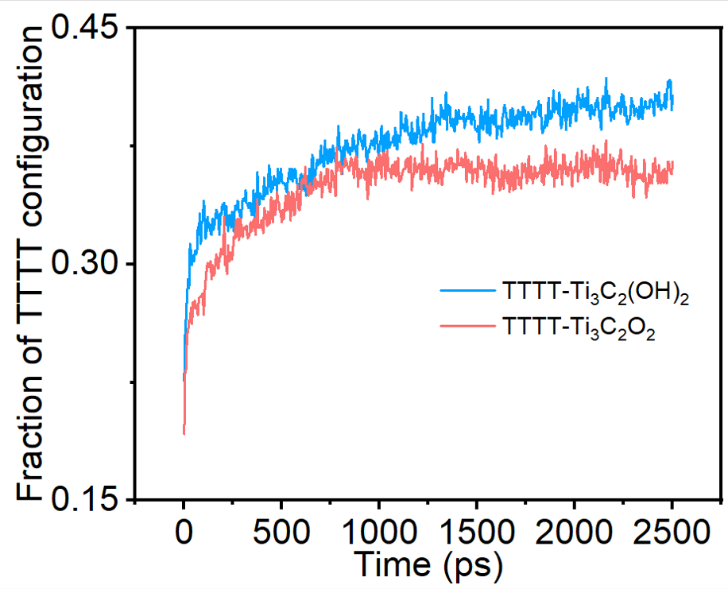


**Supplementary Fig. 7** Molecular dynamics calculated fraction of TTTT conformation of the P(VDF-TrFE) chains as a function of time.


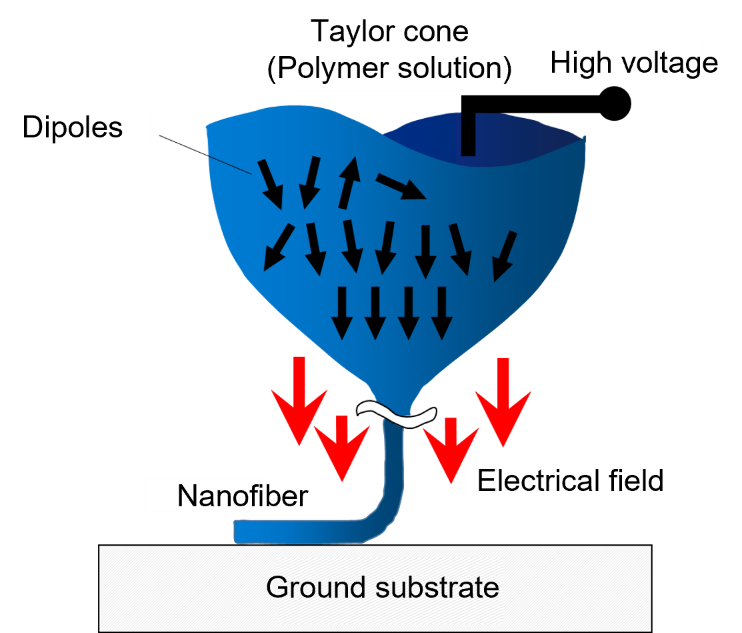


**Supplementary Fig. 8** Schematic of electrospinning.

**
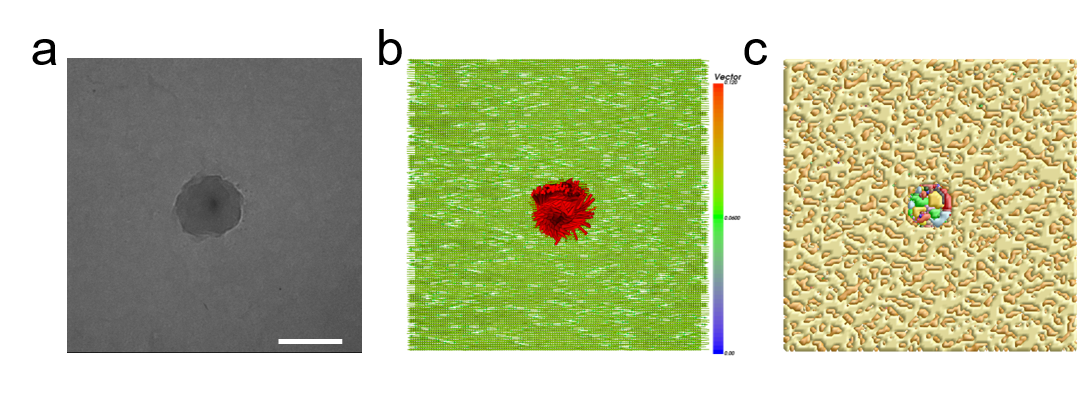
**

**Supplementary Fig. 9** Characterization and phase-field simulation of Sm-PMN-PT/MXene/PVDF composite. (a) Scanning electron microscope (SEM) image, (b) Simulated polarization and (c) Domain structure of of as-prepared Sm-PMN-PT/MXene/PVDF composite. Scale bar: 100 nm.

**
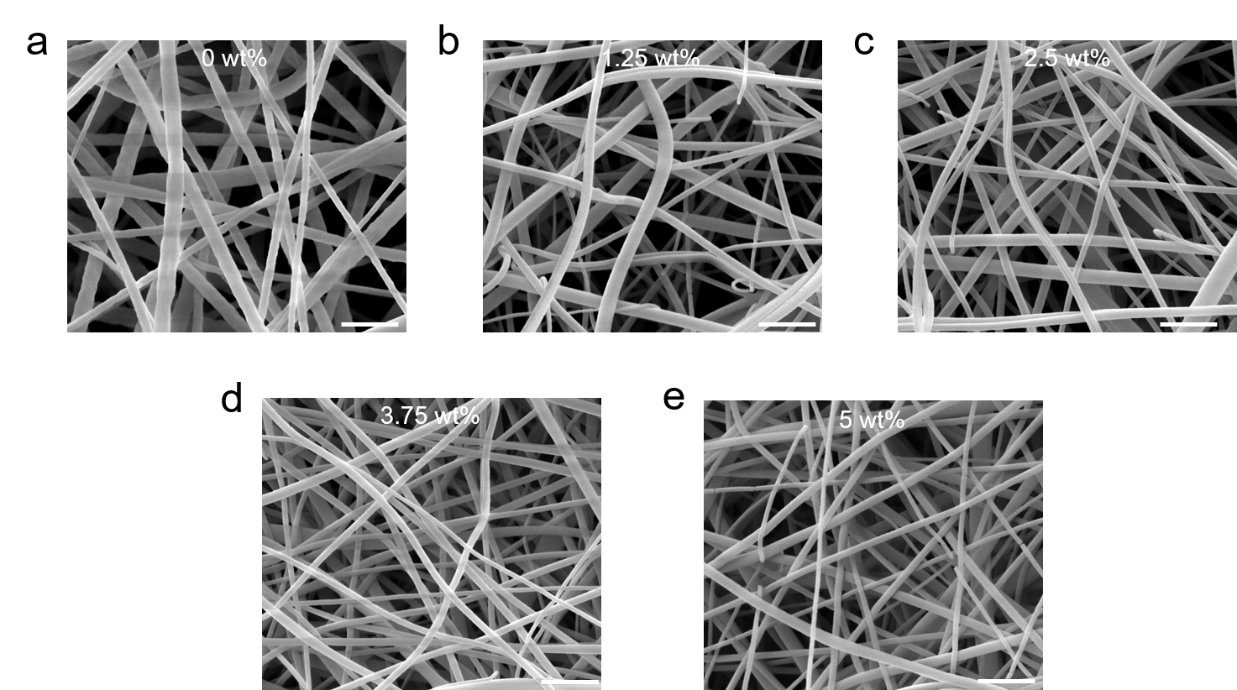
**

**Supplementary Fig. 10** SEM images of as-electrospun nanofibers by doping MXene nanosheets of (a) 0 wt%, (b) 1.25 wt%, (c) 2.5 wt%, (d) 3.75 wt% and (e) 5 wt%. Scale bar: 1 μm.


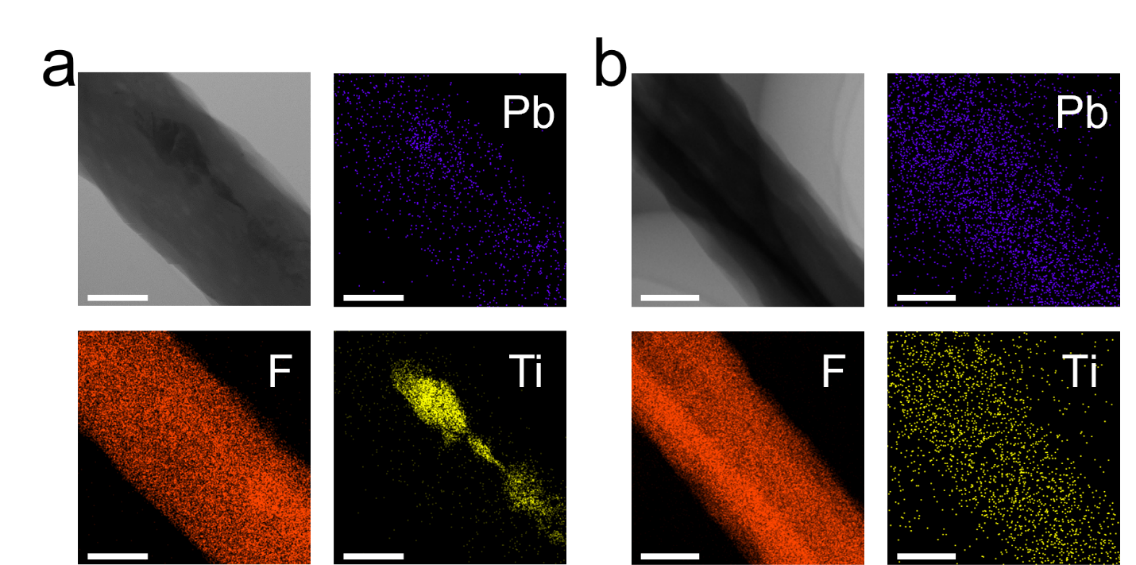


**Supplementary Fig. 11** Energy-dispersive spectrometer (EDS) mapping spectra of Pb, F, and Ti elements in the selected areas of as-prepared composite nanofibers (a) with and (b) without Sm-PMN-PT ceramic nanoparticles. Scale bars: 200 nm.

**
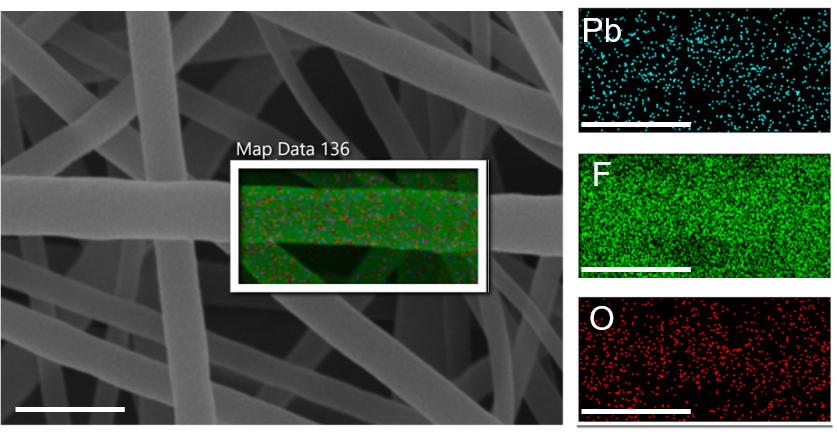
**

**Supplementary Fig. 12** Scanning electron microscope (SEM) image and Energy-dispersive spectrometer (EDS) mapping of as-prepared nanofibers. Scale bar: 500 nm.


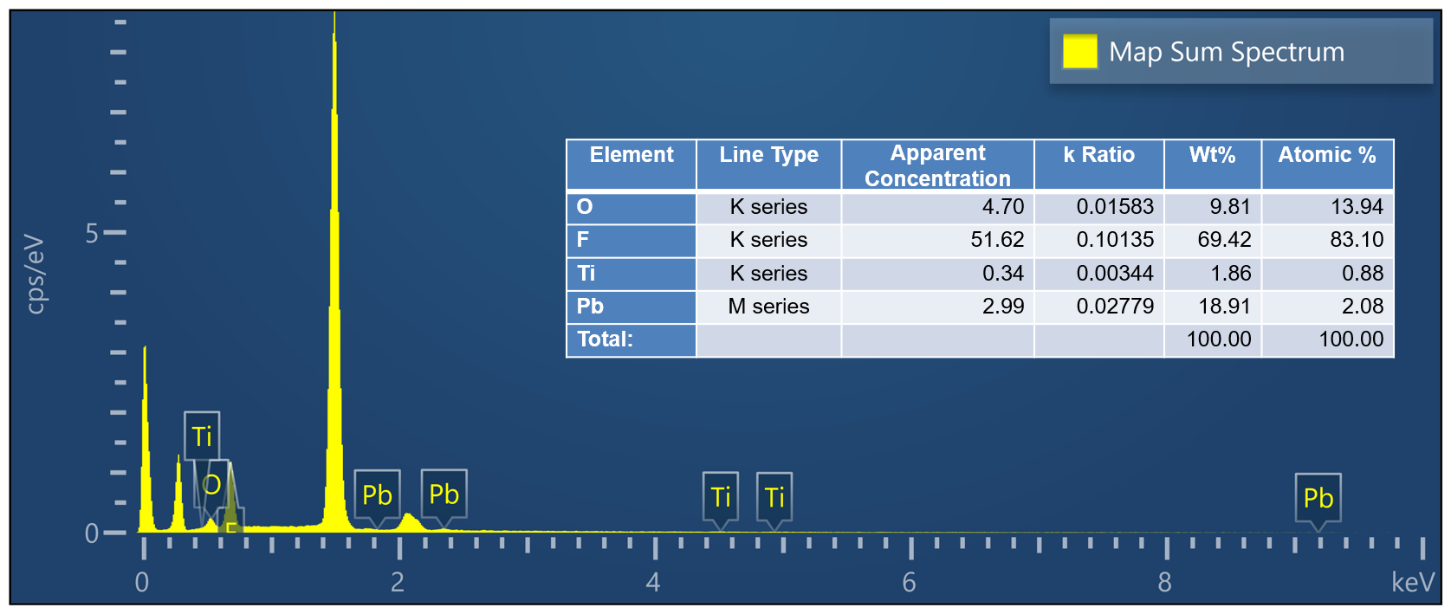


**Supplementary Fig. 13** EDS results of the prepared MPC nanofibers doped with 5 wt% doped MXene.


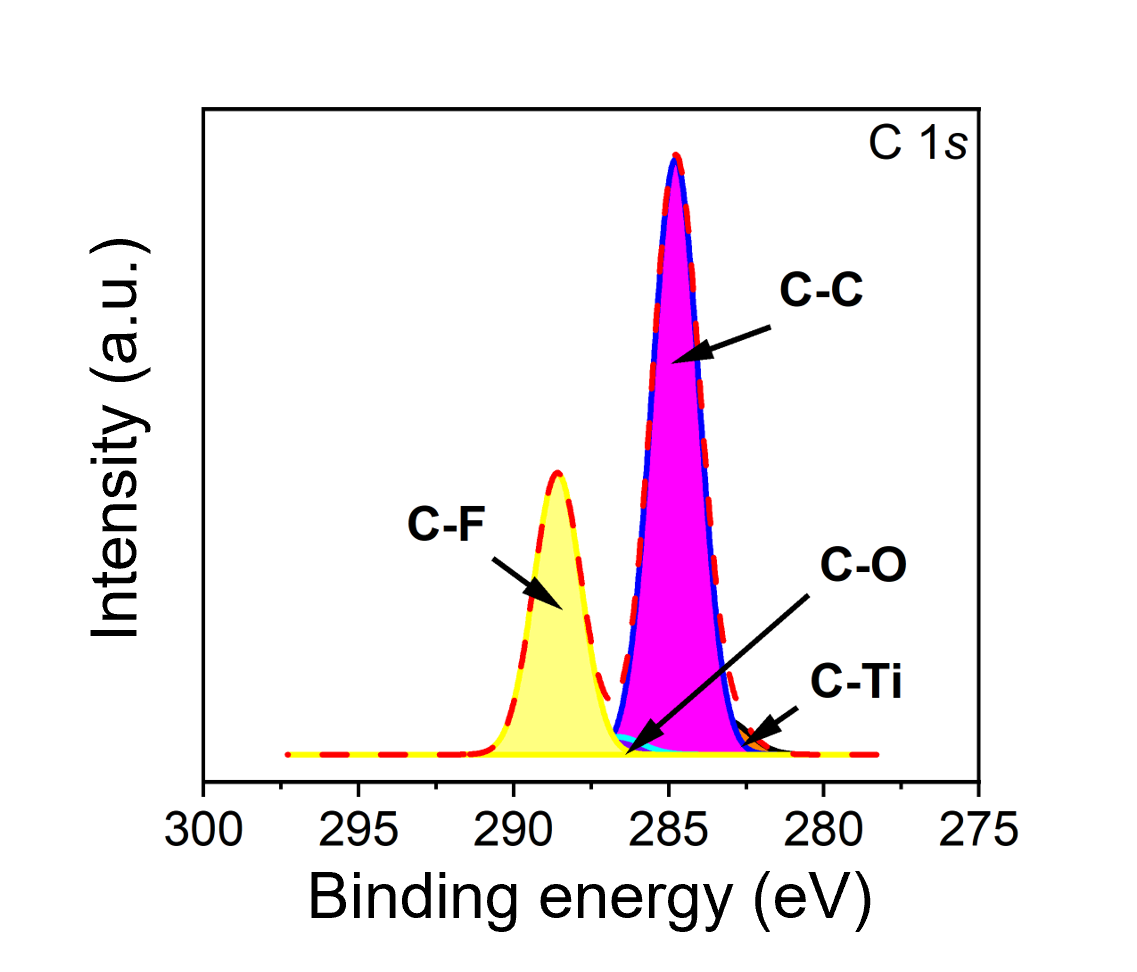


**Supplementary Fig. 14** C 1*s* X-ray photoelectron spectrometer (XPS) spectra of as-prepared undoped nanofibers.


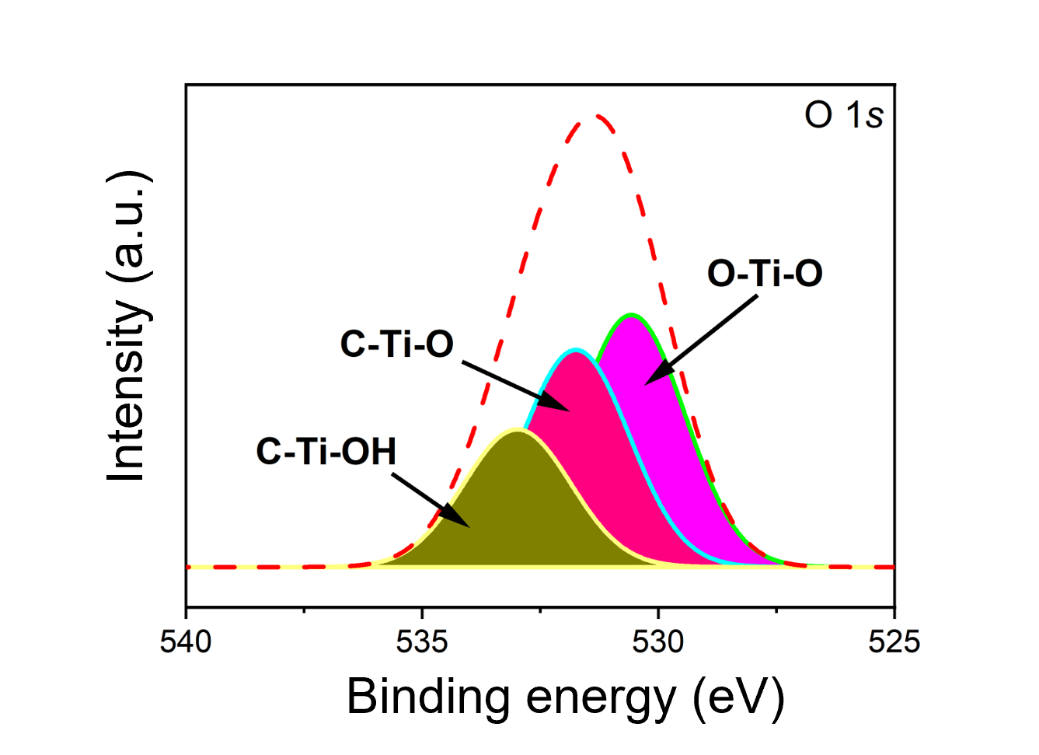


**Supplementary Fig. 15** O 1*s* X-ray photoelectron spectrometer (XPS) spectra of as-prepared MPC nanofibers.

**
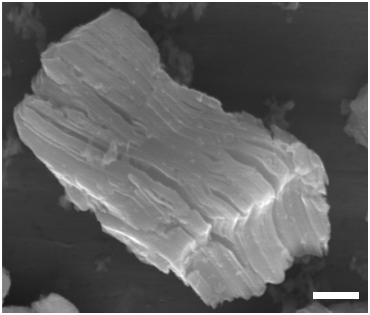
**

**Supplementary Fig. 16** SEM image of the prepared Ti_3_AlC_2_ MAX. Scale bar: 1μm.


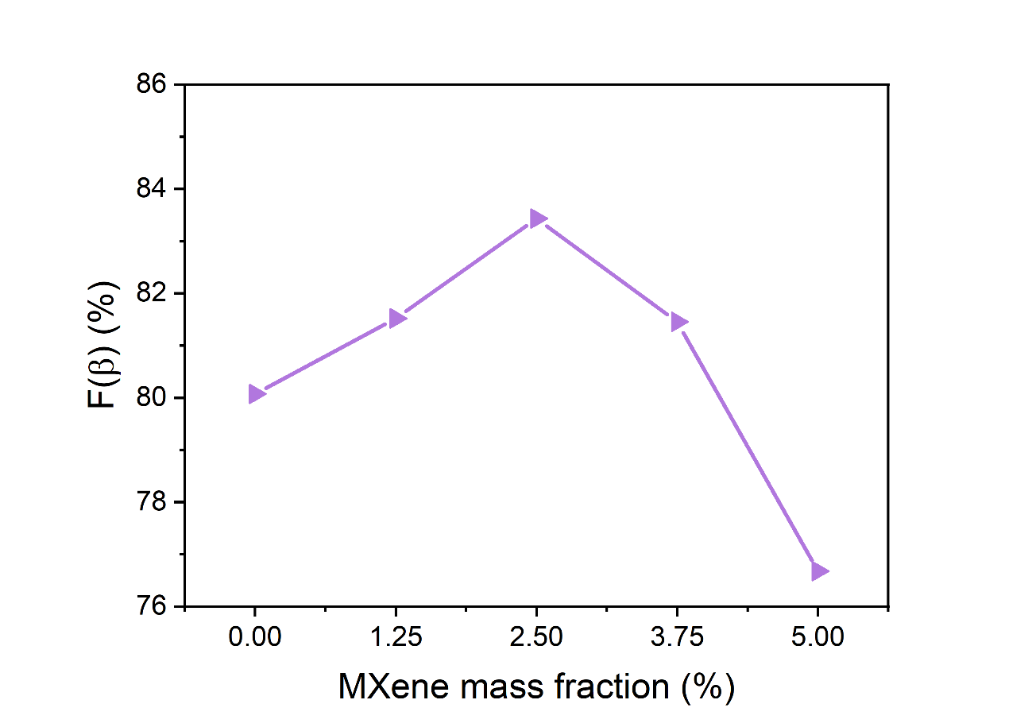


**Supplementary Fig. 17** Fraction of β phase of MPC fibers versus doped MXene mass fraction.

**
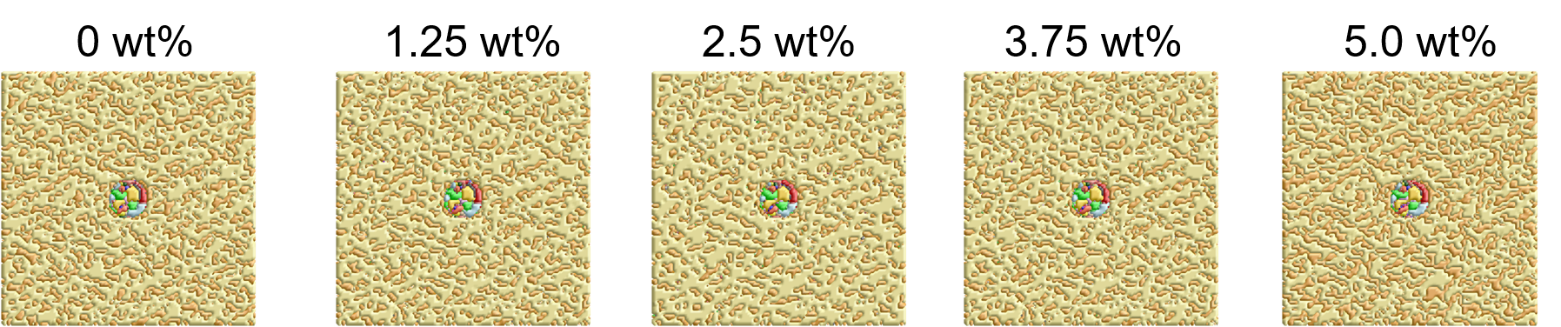
**

**Supplementary Fig. 18** Domain structures of as-synthesized composites doped with various MXene mass fractions.

**
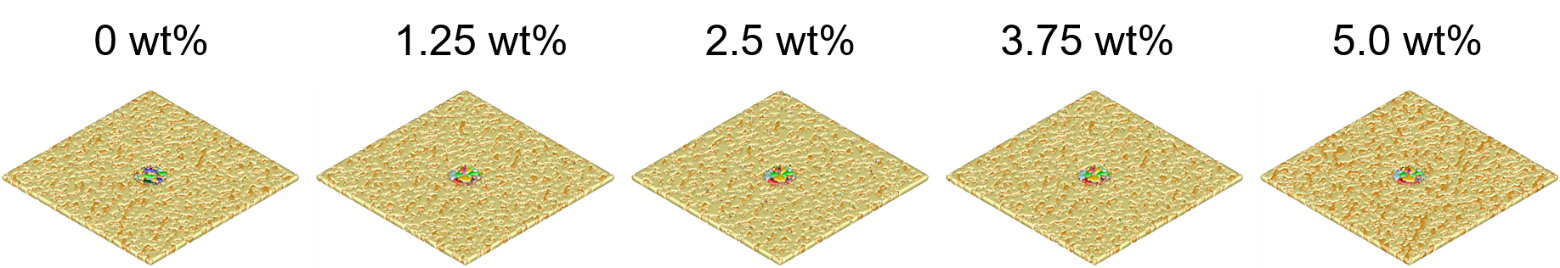
**

**Supplementary Fig. 19** 3D domain structures of as-synthesized composites doped with various MXene mass fractions.


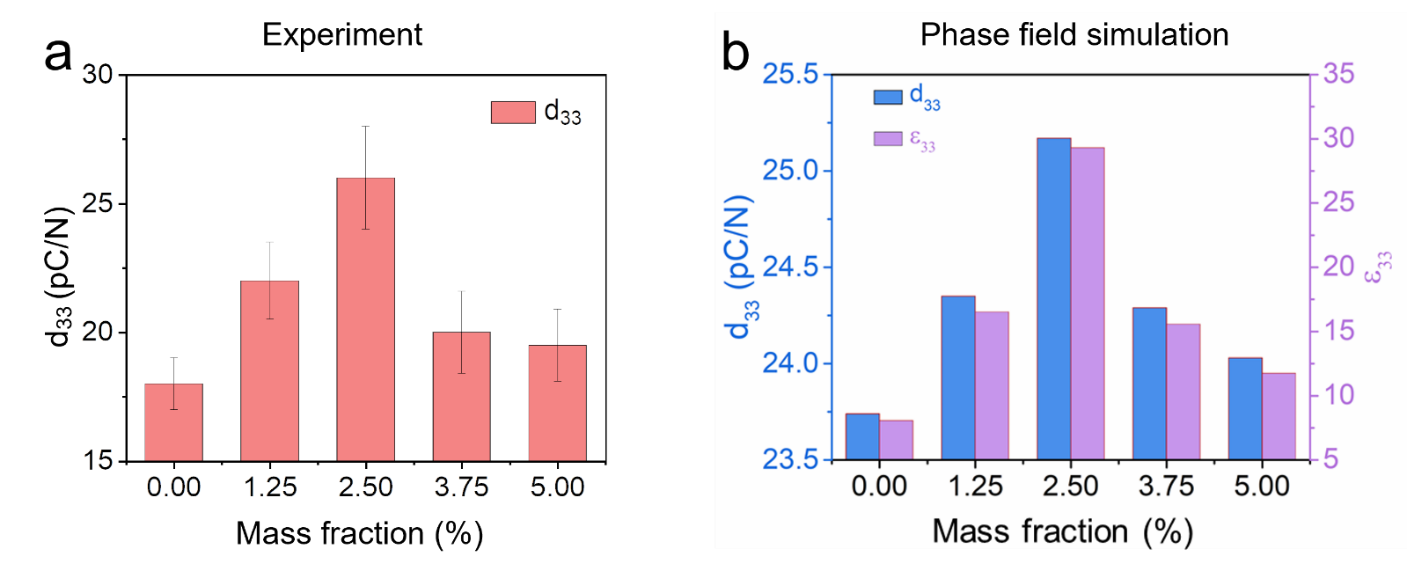


**Supplementary Fig. 20** Piezoelectric constant (*d*_33_) of electrospun films with different MXene contents. (a) *d*_33_ meter measurement, (b) Phase field simulation.

**
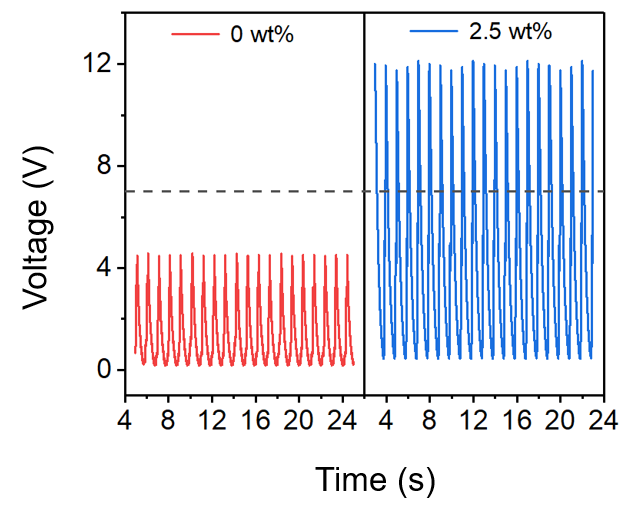
**

**Supplementary Fig. 21** Comparison in output voltage between undoped textiles and MPC textiles doped with 2.5 wt% MXene.

**
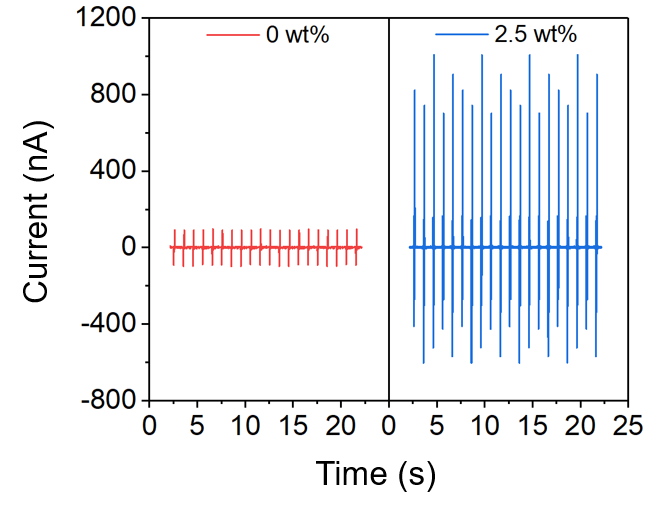
**

**Supplementary Fig. 22** Comparison in output current between undoped textiles and MPC textiles doped with 2.5 wt% MXene.

**Supplementary Note 1: Fabrication process of the prepared piezoelectric textile-based sensors.**

First, the electrospun nanofibrous composite film was tailored into small pieces of 2.5 cm × 2 cm, and sandwiched by two aluminum foil (2.5 cm × 2 cm, 1.8cm × 1.6cm) as the. A protrusion (0.45cm × 0.5cm) in the upper electrode was utilized for pasting wires. Then, conductive silver paste was employed to bond the wires and electrodes, which was placed at 30° C for 5 min for solidification. Finally, the whole device was packaged using transparent medical tape (3M) to build up the soft piezoelectric textile (PT) sensor.”


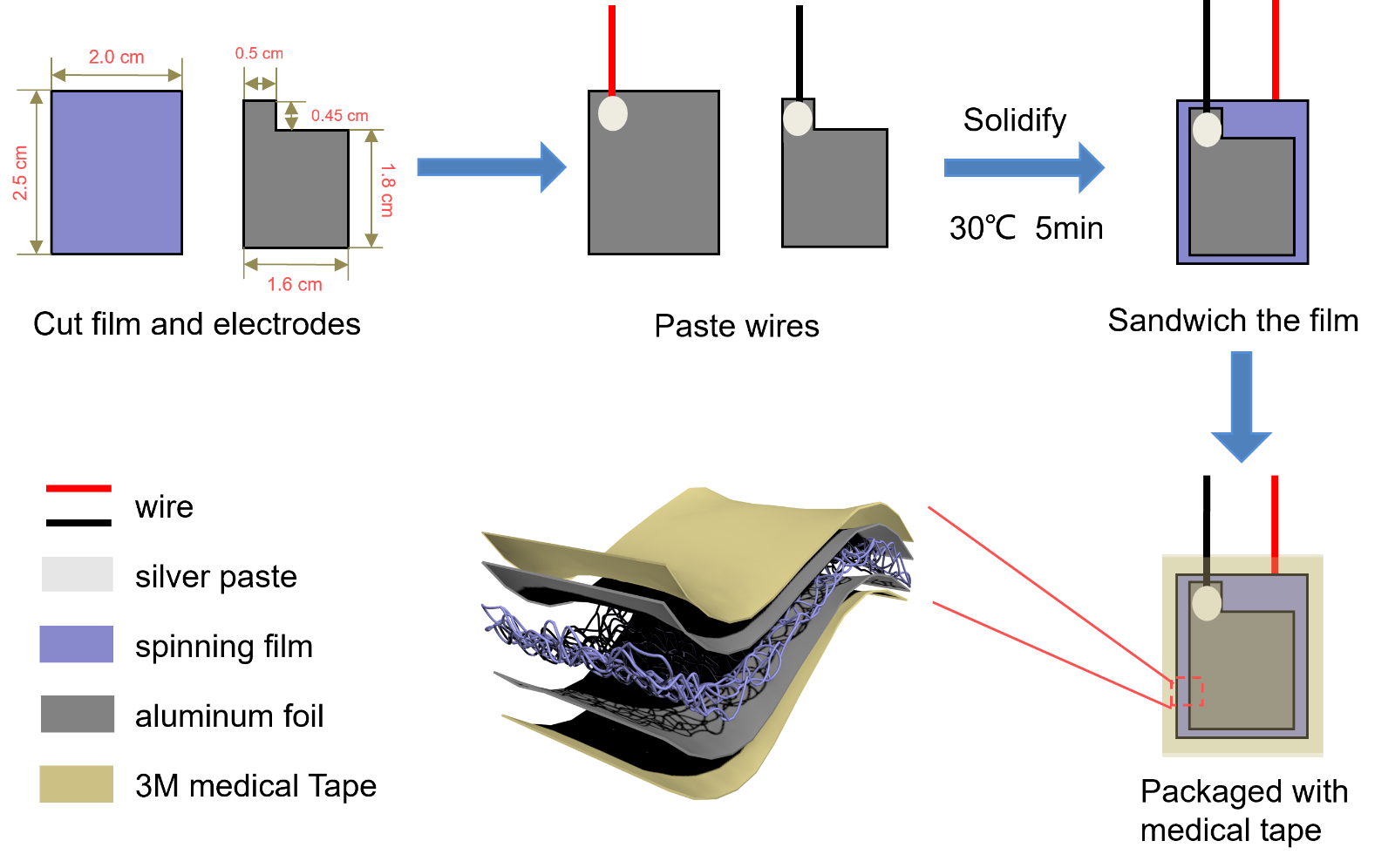


**Supplementary Fig. 23** Schematic diagram of the fabrication of the sensor.


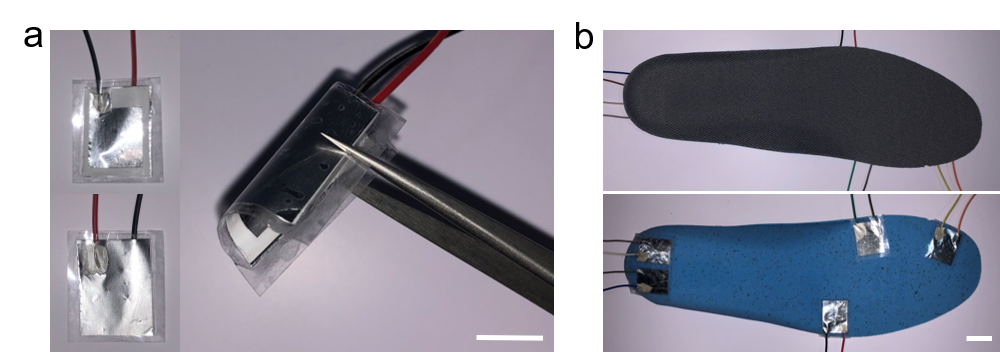


**Supplementary Fig. 24** (a) Optical image of a prepared sensor. (b) The sensor network fabricated on a conventional insole. Scale bar: 2 cm.


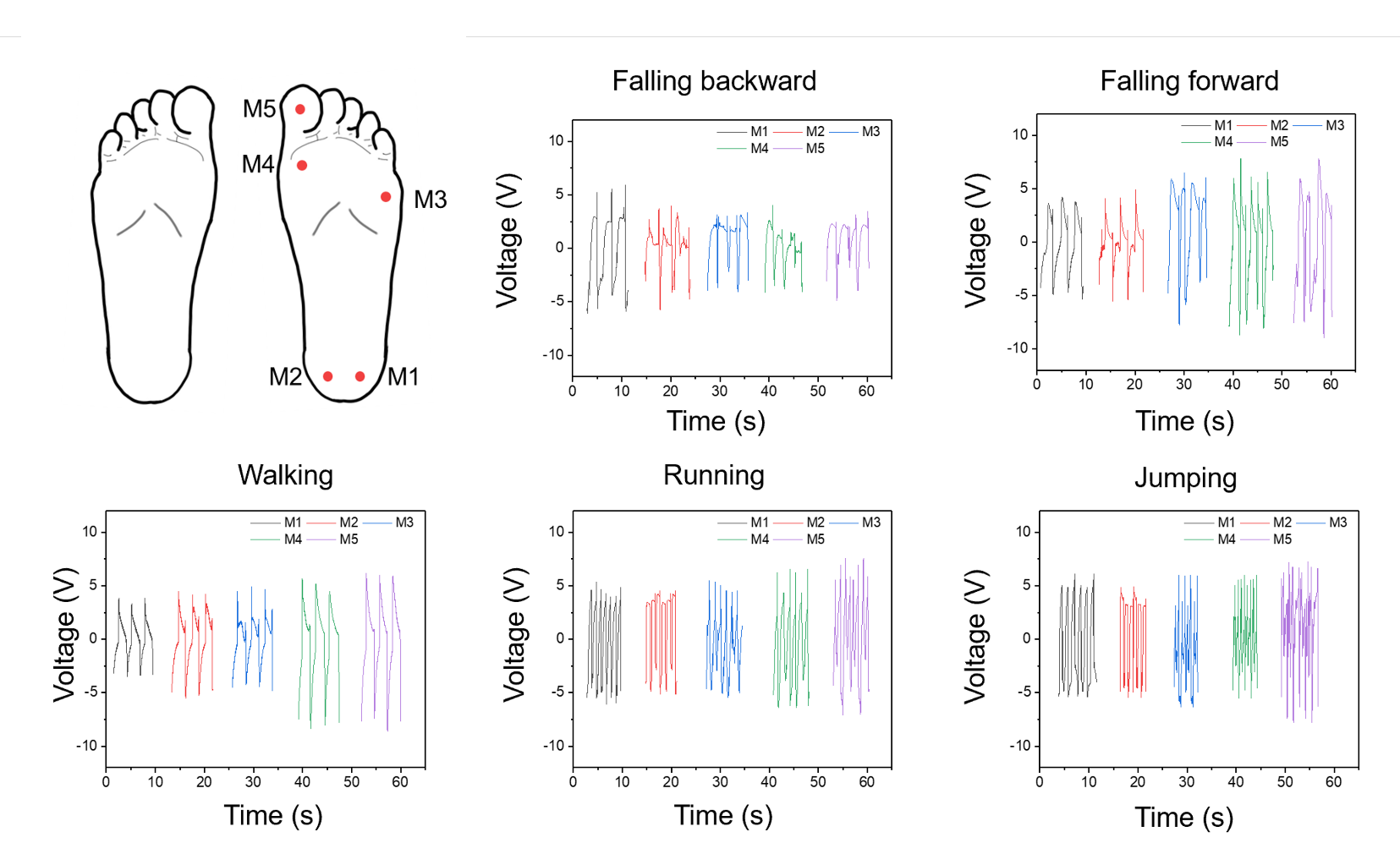


**Supplementary Fig. 25** Output voltage of as-prepared soft PT sensors doped with 2.5 wt% MXene in response to various movements.


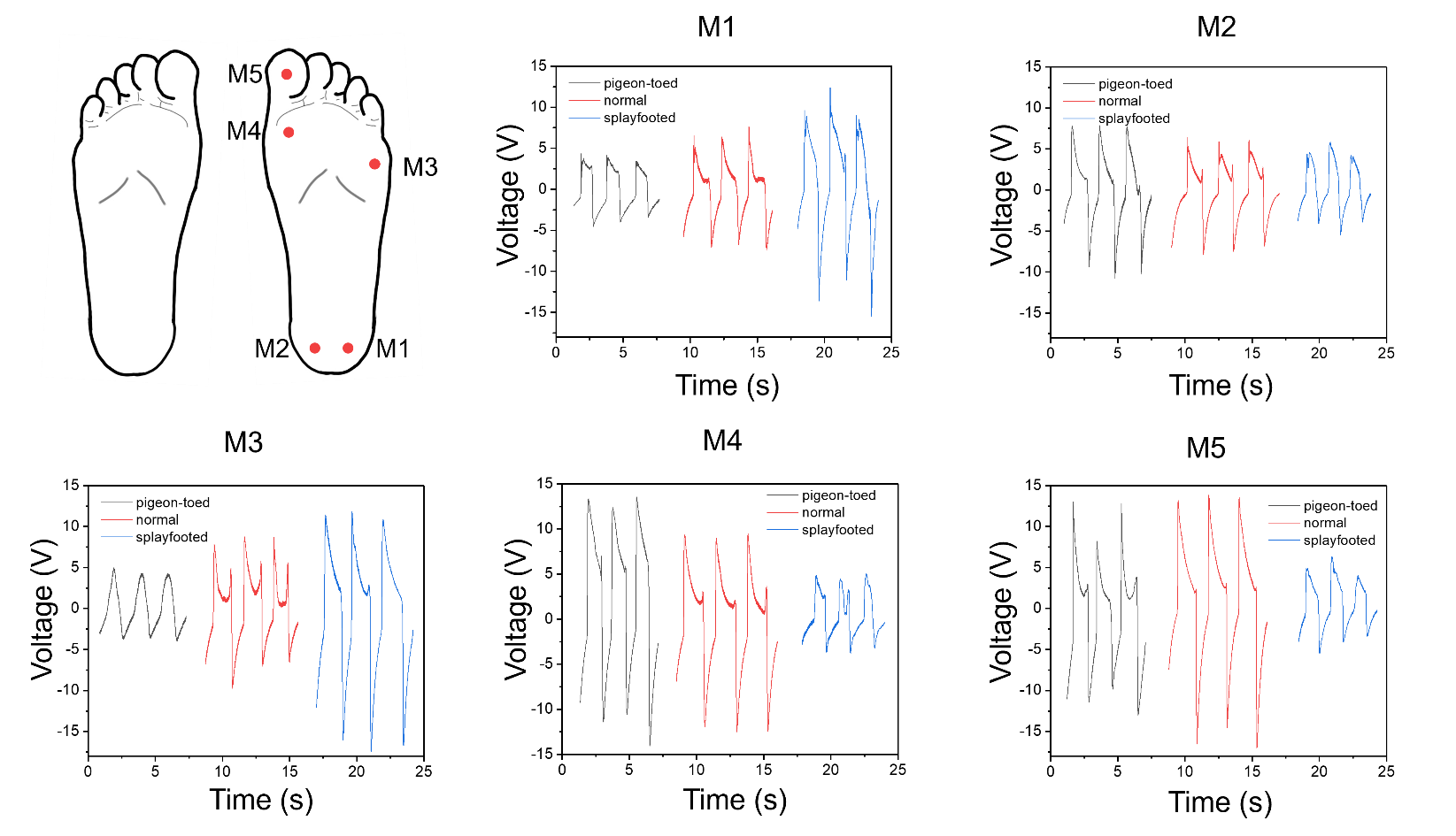


**Supplementary Fig. 26** Output voltage of as-prepared soft PT doped with 2.5 wt% MXene in response to diverse postures.


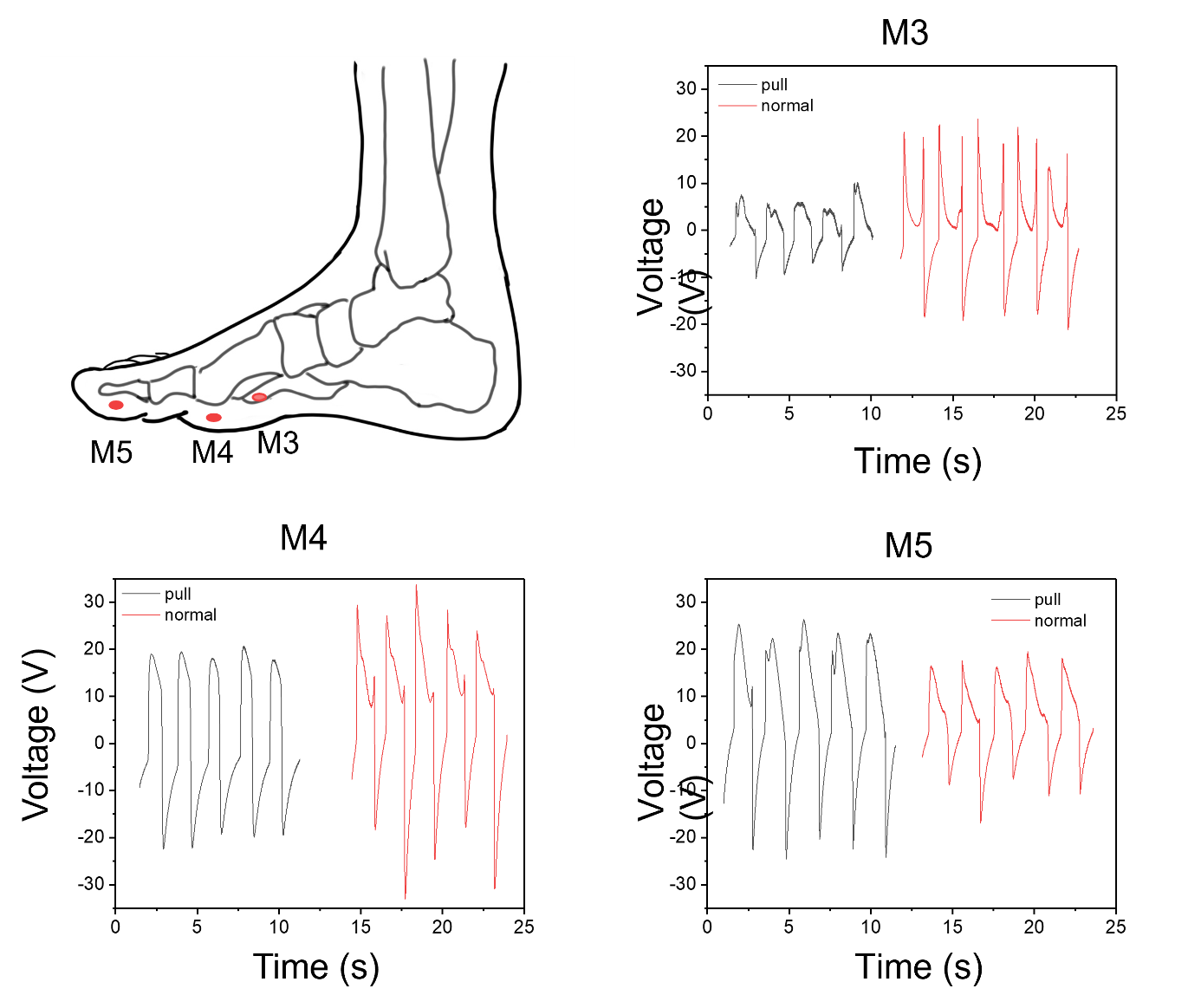


**Supplementary Fig. 27** Output voltage of as-prepared soft PT doped with 2.5 wt% MXene in response to various movements.

**Supplementary Table 1.** Parameters for the PFM characterization.

| **Parameters** | **Value** |
| --- | --- |
| Chip size | 3.4 × 1.6 × 0.3 mm |
| Reflective side | PtIr |
| Tip height: | 14-16 µm |
| Tip curvature radius | 35 nm |
| Aspect ratio | 3:1-5:1 |
| Tip side coating | PtIr (thickness 20-30 nm) |
| Cantilever length | 125±5 µm |
| Cantilever width | 30±5 µm |
| Cantilever thickness | 1.5-2.5 µm |
| Resonant frequency | 87-230 kHz |
| Force constant | 1.45-15.1 N/m |

**Supplementary Table 2.** Material constants of Sm-PMN-PT used in phase-field simulations of the domain structure.

| **Coefficients** | **Value** | **Units** |
| --- | --- | --- |
| α_1_ | (2.583×T-1204) ×10^5^ | C^-2^m^2^N |
| α_11_ | (-0.3775×T+304.2) ×10^5^ | C^-2^m^2^N |
| α_12_ | 1.085×10^8^ | C^-2^m^2^N |
| α_111_ | 2.57×10^9^ | C^-4^m^6^N |
| α_112_ | 6.95×10^9^ | C^-4^m^6^N |
| α_123_ | 1.313×10^10^ | C^-4^m^6^N |
| Q_11_ | 0.084 | C^-2^m^4^ |
| Q_12_ | -0.025 | C^-2^m^4^ |
| Q_44_ | 0.035 | C^-2^m^4^ |
| s_11_ | 9.43×10^-12^ | m^2^/N |
| s_12_ | -1.68×10^-12^ | m^2^/N |
| s_44_ | 3.509×10^-11^ | m^2^/N |

**Supplementary Table 3.** Material constants of PVDF used in phase-field simulations of the domain structure.

| **Coefficients** | **Value** | **Units** |
| --- | --- | --- |
| α_1_ | 5.647×10^9^ | C^-2^m^2^N |
| α_2_ | 5.647×10^9^ | C^-2^m^2^N |
| α_3_ | 1.412×(T-315) ×10^7^ | C^-2^m^2^N |
| α_33_ | -1.842×10^11^ | C^-4^m^6^N |
| α_333_ | 2.585×10^13^ | C^-6^m^10^N |
| Q_11_ | -8.5 | C^-2^m^4^ |
| Q_12_ | 0 | C^-2^m^4^ |
| Q_44_ | 0 | C^-2^m^4^ |
| s_11_ | 4×10^-10^ | m^2^/N |
| s_12_ | 1.11×10^-9^ | m^2^/N |
| s_44_ | 1.25×10^-9^ | m^2^/N |

**Supplementary Note 2.** Materials constants of Sm-PMN-PT and PVDF used in calculating the effective properties of the piezoelectric composites.

**Sm-PMN-PT**

Relative dielectric permittivity **ε***_r_*

$$\left[ \begin{matrix} \text{9184} & & \\ & \text{9184} & \\ & & \text{9184} \end{matrix} \right]$$

Piezoelectric coefficient **d**

$\left[ \begin{matrix} \text{0} & \text{0} & \text{0} & \text{0} & \text{1241} & \text{0} \\ \text{0} & \text{0} & \text{0} & \text{124}\text{1} & \text{0} & \text{0} \\ \text{-446} & \text{-446} & \text{1050} & \text{0} & \text{0} & \text{0} \end{matrix} \right]$ (pC/N)

Elastic stiffness **c**

$\left[ \begin{matrix} \text{207.3} & \text{166.4} & \text{109.3} & \text{0} & \text{0} & \text{0} \\ \text{166.4} & \text{207.3} & \text{109.3} & \text{0} & \text{0} & \text{0} \\ \text{109.3} & \text{109.3} & \text{110.1} & \text{0} & \text{0} & \text{0} \\ \text{0} & \text{0} & \text{0} & \text{19.9} & \text{0} & \text{0} \\ \text{0} & \text{0} & \text{0} & \text{0} & \text{19.9} & \text{0} \\ \text{0} & \text{0} & \text{0} & \text{0} & \text{0} & \text{20.4} \end{matrix} \right]$ (GPa)

**PVDF**

Relative dielectric permittivity **ε***_r_*

$$\left[ \begin{matrix} \text{12} & & \\ & \text{12} & \\ & & \text{12} \end{matrix} \right]$$

Piezoelectric coefficient **d**

$\left[ \begin{matrix} \text{0} & \text{0} & \text{0} & \text{0} & \text{19.3} & \text{0} \\ \text{0} & \text{0} & \text{0} & \text{20.1} & \text{0} & \text{0} \\ \text{13.6} & \text{19.4} & \text{29.7} & \text{0} & \text{0} & \text{0} \end{matrix} \right]$ (pC/N)

Elastic stiffness **c**

$\left[ \begin{matrix} \text{1.69} & \text{0.87} & \text{0.87} & \text{0} & \text{0} & \text{0} \\ \text{0.87} & \text{1.69} & \text{0.87} & \text{0} & \text{0} & \text{0} \\ \text{0.87} & \text{0.87} & \text{1.69} & \text{0} & \text{0} & \text{0} \\ \text{0} & \text{0} & \text{0} & \text{0.41} & \text{0} & \text{0} \\ \text{0} & \text{0} & \text{0} & \text{0} & \text{0.41} & \text{0} \\ \text{0} & \text{0} & \text{0} & \text{0} & \text{0} & \text{0.41} \end{matrix} \right]$ (GPa)
