# Supplementary material for: High-performance piezoelectric composites via β phase programming
Source: Nat Commun. 2022 Aug 18;13:4867. doi: 10.1038/s41467-022-32518-3 (PMC9388583; doi:10.1038/s41467-022-32518-3)
Supplement: Supplementary file 2 — Description of Additional Supplementary Files [file 41467_2022_32518_MOESM2_ESM.pdf]

Description of Additional Supplementary Files

Supplementary Movie 1: Generation of electric signals corresponding to biomechanical motions.
